# Spatial prediction of the concentration of selenium (Se) in grain across part of Amhara Region, Ethiopia

**DOI:** 10.1016/j.scitotenv.2020.139231

**Published:** 2020-09-01

**Authors:** D. Gashu, R.M. Lark, A.E. Milne, T. Amede, E.H. Bailey, C. Chagumaira, S.J. Dunham, S. Gameda, D.B. Kumssa, A.W. Mossa, M.G. Walsh, L. Wilson, S.D. Young, E.L. Ander, M.R. Broadley, E.J.M. Joy, S.P. McGrath

**Affiliations:** a Centre for Food Science and Nutrition, Addis Ababa University, P.O. Box 1176, Addis Ababa, Ethiopia; b School of Biosciences, University of Nottingham, Sutton Bonington, Leicestershire LE12 5RD, UK; c Department of Sustainable Agriculture Sciences, Rothamsted Research, Harpenden, Hertfordshire AL5 2JQ, UK; d International Crop Research Institute for the Semi-Arid Tropics, ILRI Sholla Campus, P.O. Box 5689, Addis Ababa, Ethiopia; e International Maize and Wheat Improvement Center, ILRI Sholla Campus, P.O. Box 5689, Addis Ababa, Ethiopia; f Center for International Earth Science Information Network, The Earth Institute, Columbia University, 61 Route 9W, Geoscience Building Suite 201, Palisades, New York 10964, USA; g British Geological Survey, Keyworth, Leicestershire NG12 5GG, UK; h Faculty of Epidemiology and Population Health, London School of Hygiene & Tropical Medicine, Keppel Street, London WC1E 7HT, UK.

**Keywords:** Selenium, Micronutrients, Hidden hunger, Teff, Wheat, Geostatistics

## Abstract

Grain and soil were sampled across a large part of Amhara, Ethiopia in a study motivated by prior evidence of selenium (Se) deficiency in the Region's population. The grain samples (teff, *Eragrostis tef*, and wheat, *Triticum aestivum*) were analysed for concentration of Se and the soils were analysed for various properties, including Se concentration measured in different extractants. Predictive models for concentration of Se in the respective grains were developed, and the predicted values, along with observed concentrations in the two grains were represented by a multivariate linear mixed model in which selected covariates, derived from remote sensor observations and a digital elevation model, were included as fixed effects. In all modelling steps the selection of predictors was done using false discovery rate control, to avoid over-fitting, and using an α-investment procedure to maximize the statistical power to detect significant relationships by ordering the tests in a sequence based on scientific understanding of the underlying processes likely to control Se concentration in grain. Cross-validation indicated that uncertainties in the empirical best linear unbiased predictions of the Se concentration in both grains were well-characterized by the prediction error variances obtained from the model. The predictions were displayed as maps, and their uncertainty was characterized by computing the probability that the true concentration of Se in grain would be such that a standard serving would not provide the recommended daily allowance of Se. The spatial variation of grain Se was substantial, concentrations in wheat and teff differed but showed the same broad spatial pattern. Such information could be used to target effective interventions to address Se deficiency, and the general procedure used for mapping could be applied to other micronutrients and crops in similar settings.

## Highlights

• Joint soil and crop sampling was undertaken across Amhara Region, Ethiopia.
• Statistical modelling of the resulting data incorporated extensive covariates.
• Spatial predictions were made of grain selenium concentration.
• These predictions, and their uncertainties, are presented as maps.
• The maps show the probability that grain provides adequate dietary Se.

## Graphical abstract

**Figure:**
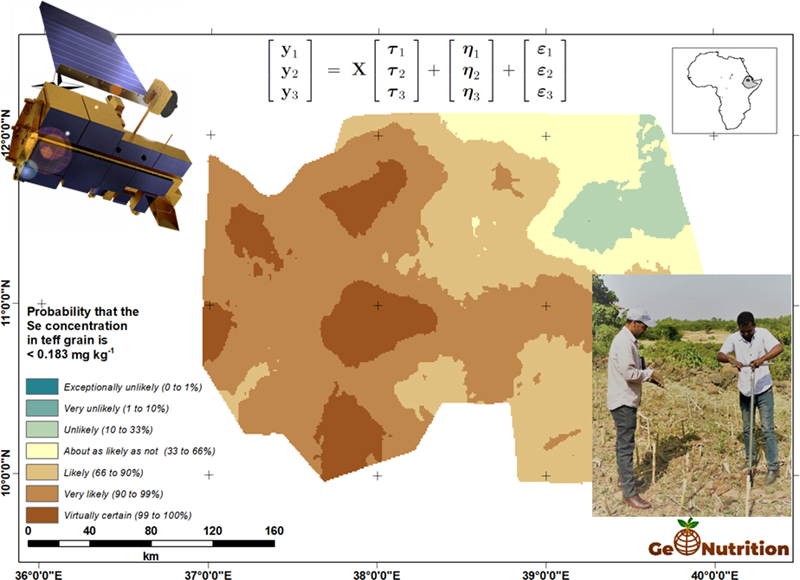

## 1 Introduction

Mineral micronutrient deficiencies (MNDs) are widespread in sub-Saharan Africa (SSA), especially among women and children (Joy et al., 2014; Kumssa et al., 2015; Schmidhuber et al., 2018; Smith et al., 2016). These deficiencies, sometimes called ‘hidden hunger’, are a critical obstacle to the United Nations' second Sustainable Development Goal (SDG2, ‘Zero Hunger’), to ‘achieve food security and improved nutrition’ by 2030 (Gödecke et al., 2018).

There are multiple and complex causes of MNDs, including poor dietary intake and bioavailability as well as nutrient losses due to factors such as infection (Caulfield et al., 2006). In SSA many mineral MNDs arise from restricted soil-to-crop transfer of micronutrients, due to soil conditions, exacerbated by poor dietary diversity including a paucity of animal-source foods (Hurst et al., 2013; Joy et al., 2014, Joy et al., 2015; Manzeke et al., 2019; Phiri et al., 2019).

There are various interventions available to address MNDs, including biofortification through crop breeding. There have been notable successes by HarvestPlus and the Consultative Group for International Agricultural Research (CGIAR) to develop staple crops with increased grain concentration of iron (Fe) in SSA, and zinc (Zn) in South Asia (Gregory et al., 2017; Khokhar et al., 2018; Velu et al., 2012). However, the alleviation of multiple mineral MNDs in SSA is likely to require combined approaches including dietary diversification, food fortification, and the use of micronutrient-enriched fertilisers (agronomic biofortification). There are precedents for using Se containing fertilisers at national scale in Finland, where Se agronomic biofortification has been continually used in crop production since 1984 (Chilimba et al., 2012).

Effective intervention to address MNDs requires reliable information to support decision making at national and subnational scales. Such information is relatively scarce in SSA. Little is known about how soil-to-crop transfers of minerals and intake of minerals into food systems vary spatially, and there is a lack of reliable biomarkers of micronutrient status to identify where particular micronutrients are in deficit and where they are adequate. It has been shown that considerable efficiencies could be achieved if particular interventions were targeted and tailored to local conditions (Vosti et al., 2015), and so this lack of information limits the ability of policy makers to design effective responses.

Some of the richest disaggregated data for Se in SSA are found in Ethiopia and Malawi although even these data are relatively sparse. In both countries the variation of population nutrient status can be attributed in part to local soil conditions, and to other landscape and socio-economic factors (Gashu et al., 2016a, Gashu et al., 2016b; Hurst et al., 2013; Phiri et al., 2019, Phiri et al., 2020). In Malawi, Phiri et al., 2019, Phiri et al., 2020 showed marked spatial national-scale variation in the Se status of women of reproductive age, and the spatial patterns were consistent with previous surveys of the Se concentration in soil and maize grain (Chilimba et al., 2011), and with smaller cross-sectional studies of Se intake and status (Hurst et al., 2013).

Comparable information on the spatial variation of Se status among the Ethiopian population, and contributing factors, have not yet been reported at national scale. The overall prevalence of Se deficiency is likely to be large. For example, Gashu et al. (2016a) identified widespread Se deficiency, based on a large-scale survey of the serum Se status of children in the Amhara Region (east Gojjam and west Gojjam, south and north Wollo, north Gonder and Waghera Districts). Approximately 55% of these were deficient. They hypothesized that Se deficiency risks were linked to soil and/or landscape features (Gashu et al., 2016a). Reliable data on the Se status of soils and crops in Ethiopia, and elsewhere in SSA, are lacking (Ligowe et al., 2020). Sillanpää and Jansson (1992) reported the Se status of 126 soils and co-located plants (wheat or maize) in Ethiopia. However, their sampling was not designed to provide spatial coverage, and did not include the important staple crop teff. Sillanpää and Jansson (1992) concluded that the Se status of crops in Ethiopia was generally satisfactory but that localized deficiency may exist. Ligowe et al. (2020) re-analysed these data. Topsoil Se concentration, following acid ammonium acetate-EDTA universal extraction, ranged from <5–32 μg L^−1^, and there was no evidence for relationships between concentrations of Se in soil and concentrations in maize or wheat. In summary, there is evidence for Se deficiency in parts of Amhara Region, and preliminary evidence of variation in Se concentration in soil, but further focussed sampling is necessary to understand this variation, and its possible relationship to Se concentration in crops. On the basis of the results of Gashu et al. (2016a), who show that there are substantial rates of Se deficiency among children in Amhara Region, this is an appropriate area in which to undertake such a study.

The objective of the study reported here was to examine evidence for the spatial variation of Se concentration in cereal staple crops across part of the Amhara Region of Ethiopia. In particular we wished to examine how field surveys of crop and soil, along with additional spatial information, could be used to make reliable spatial predictions of Se concentration in grain, with attached measures of uncertainty. Our hypothesis was that at least some of the observed variation in Se status of grain can be accounted for by the effects of variation in soil properties, and so that soil information can be used, along with direct measurements of Se in grain, to make better spatial predictions of grain Se concentration than could otherwise be produced. Such maps could provide a basis for understanding patterns of Se deficiency in the population, and for identifying areas where such deficiencies might be expected, and where particular interventions might be most appropriate because of the poor local Se status of staple crops. From previous dietary data analyses (Gashu et al., 2016b), teff (*Eragrostis tef*) and wheat (*Triticum aestivum*) are the two dominant cereal crops in this region. Teff was the most widely-consumed cereal, eaten by 76% of children in the previous 24 h. We therefore focus in this study on mapping the concentration of Se in teff and in wheat grain.

This mapping task is a substantial challenge for several reasons. First, data on grain Se status, even in a focussed survey, are inevitably sparse, and there is likely to be considerable variation in these data at multiple scales. Second, while soil properties can be measured at any sample site, grain Se can only be measured from sites where that particular grain was growing. We therefore have a mixture of collocated observations of soil properties and the target grain, and non-collocated observations of soil properties and the non-target grain. Covariates, including remotely sensed data, may help the process of spatial prediction, but we require robust methods to select appropriate covariates for any prediction task. Finally, the predictions that are made have inevitable uncertainty. If they are to be useful then we must be able to quantify this uncertainty and to communicate it appropriately to the relevant stakeholders. Given these considerations we decided to use the spatial linear mixed model (LMM) for the analysis and prediction of data on grain Se, soil properties and associated covariates (Cressie, 1993). Specifically we considered a multivariate version of the model (Marchant and Lark, 2007) which allows us to combine collocated and non-collocated data. The empirical best linear unbiased prediction (E-BLUP), based on the fitted model, has an associated prediction error distribution, and on the basis of this we were able to quantify uncertainties in the prediction relative to threshold concentrations of interest, and to use strategies to communicate this uncertainty which have been used elsewhere (Lark et al., 2014, Lark et al., 2019; Mastrandrea et al., 2010).

## 2 Materials and methods

### 2.1 Sampling

The objective of field sampling was to support spatial prediction of grain Se concentration. To this end it was neither necessary nor desirable to sample independently and at random. The objective was to obtain samples that gave reasonable spatial coverage over the target sample frame, with a proportion of sample points at a short distance from the basic sample set to support the estimation of a spatial LMM (Lark and Marchant, 2018). The sample frame was defined in terms of the objectives and constraints of the task. First, the sample frame was constrained to sites within Amhara Region where the probability that the land was in agricultural use equalled or exceeded 0.9. This was based on predictions produced on a 500-m grid by the AfSIS project (Walsh et al., 2019) using a combination of interpretation of high-resolution satellite imagery by trained observers and machine learning methods applied to multiple covariates derived from remote sensor data and digital elevation models (AfSIS, 2015). The mapped probabilities of cropping used here are shown in Fig. S1 of the supplementary material. Second, the frame was constrained to include only those sites from a 500-m grid, that fell within 2.5 km of a known road. A map indicating nodes on a 500-m grid (with the same origin as the agricultural land use grid) which met this requirement was prepared. Information on the distribution of roads was taken from OpenStreetMap (OpenStreetMap contributors, 2017). It is acknowledged that this constraint introduces a possible bias into predictions made at sites outside the defined sample frame, and the predictions must be interpreted with it in mind. However, without such a constraint it would not have been possible to visit all sample sites across the region of interest in the time available.

Having defined the sampling frame, a total of 475 sample locations were selected with every 500-m grid node within the sampling frame allocated an equal prior inclusion probability. This was done using the lcube package from the BalancedSampling library for the R platform (R Core Team, 2017; Grafström and Lisic, 2016). This implements the cube method of Deville and Tillé (2004), which allows one to sample honouring specified inclusion probabilities while aiming for balance and spread with respect to specified covariates. In this case sample sites were selected for spatial balance, which entails that the mean coordinates of sample sites are close to the mean coordinates of all points in the sample frame) and spatial spread (which ensures that the observations are spread out rather than clustered with respect to spatial coordinates), see Grafström and Schelin (2014). Once these sites were chosen a subset of 25 was selected, again to achieve spatial spread. Each of these 25 sites were earmarked for a second field sample site at a nearby location (see next section). As stated above, the inclusion of these extra close-paired sites was done to support estimation of parameters of the spatial LMM, following Lark and Marchant (2018).

### 2.2 Field sampling

Sampling was done by teams who undertook initial training to standardize procedures. Each team aimed to visit around 5 sample sites per day. The day's sample sites were uploaded onto a tablet PC and a GPS device as a waypoint list. They were also printed on a paper map. The team would navigate to the target sampling point using the paper map, and then using the GPS over the last few kilometres. At the sample site the team would find the nearest field with a mature cereal crop within a 1-km radius, and would request permission to sample from the farmer. If a field with a standing mature cereal crop was not present then the team would talk with local farmers to identify a field where the crop had recently been harvested. If permission could be obtained to sample both this field and the stored grain which had been harvested from it, then the field would be selected. If this procedure failed then the team would look further than 1 km from the target site for an alternative. If one could not be found then the target site was abandoned. In practice it was possible in all cases to sample a standing crop, grain from field stacks or, in a few cases, grain which had been moved from the field to a store.

Samples were taken from a 100-m^2^ (0.01-ha) circular plot in the selected field. This was centred as close as possible to the middle of the field unless this appeared unrepresentative with respect to disease or crop damage. Five sub-sample sites were located, the first at the centre of the plot. Two sub-sample points were selected at locations on a line through the plot centre along the crop rows, and two on a line orthogonal to the first through the plot centre (see Fig. S2 in the supplementary material). Note that these four sub-sample points lie on the circumferences of 25-m^2^, 50-m^2^, 75-m^2^ and 100-m^2^ subplots with a common centre where the first sub-sample was collected. The central sampling location was fixed between crop rows, and the ‘long’ axis of the sample array (with sample locations at 5.64 and 4.89 m) was oriented in the direction of crop rows with the ‘short axis’ perpendicular to the crop rows (see Fig. S2 in the Supplementary Material).

A single soil subsample was collected at each of the five sub-sample points with a Dutch auger with a flight of length 150 mm and diameter 50 mm. The teams were trained to take care to insert the auger vertically and to the precise depth of one flight. Any plant material adhering to the auger was carefully removed, and the five sub-samples stored in a single bag.

Crop samples were taken close to each augering position. A grain sub-sample was collected at each site, taking care to avoid any contamination of the grain with soil. If the crop was in field stacks then a sub-sample, comprising five heads of grain, was taken from each available stack, taking material from the centre of the stack to minimize contamination by dust and soil.

At sample sites earmarked for a second ‘close-pair’ sample a duplicate field was identified where possible. Ideally this was within 500 m of the primary sample site, but a close-pair site could lie within 100–1000 m of the primary site. If such a site could not be found, then an attempt was made to find a close-pair site at the next sample location not already earmarked for a close-pair.

Photographs of sample bags and the sample site were recorded for quality assurance along with site GPS locations.

The distribution of sample points is shown on a map of Ethiopia in Fig. S3 in the supplementary material.

### 2.3 Sample preparation and laboratory analysis

#### 2.3.1 Sample preparation

The soil samples were oven-dried in their sample bags at 40^∘^C for 24 or 48 h depending on the moisture content of the soil. Preparation took place in a soil laboratory to avoid cross-contamination with grain samples in which concentrations are smaller. Any fresh plant material was removed from each sample which was then disaggregated and sieved to pass 2 mm. This material was then coned and quartered to produce sub sample splits. One such 150-g subsample was poured into a self-seal bag, labelled and shipped to the UK for analysis in the laboratories at Rothamsted Research and University of Nottingham as described below.

Grain samples were air-dried in their sample bags. All preparation was done away from sources of contamination by soil or by dust. Each sample was then ground in a coffee grinder which was wiped clean before use and after each sample with a non-abrasive cloth. A 20-g subsample of the ground material was then bagged and labelled for shipping to the University of Nottingham.

#### 2.3.2 Laboratory analysis

Crop samples were analysed for elemental composition by inductively coupled plasma mass spectrometry (ICP-MS) following microwave-assisted acid digestion in Primar Plus^TM^ grade HNO_3_ as described by Kumssa et al. (2017).

A soil sequential fractionation procedure was adapted from Mathers et al. (2017) and Shetaya et al. (2012) to provide three fractions of Si, S, Se and I nominally identified as ‘Soluble’ (0.01 M KNO_3_), ‘Adsorbed’ (0.016 M KH_2_PO_4_) and ‘Organic’ (10% TMAH). Analysis was by ICP-MS (Thermo Fisher iCAP Q) in H_2_ cell mode (Si and Se) or He cell mode with kinetic energy discrimination (I and S).

Soil pH was measured with a Jenway 3540 m, with a temperature-compensated combination pH electrode, where the soil:water suspension ratio was 1:2.5, with 60 min equilibrating time.

Acid oxalate extractable Fe, Al, Mn and P were extracted with a mixed solution of ammonium oxalate and oxalic acid at a soil: solution ratio of 1:100 (Schwertman, 1964). Samples were shaken in the dark (4 h, 20^∘^C) using a reciprocal shaker, filtered then acidified and analysed by inductively coupled plasma optical emission spectrometry (ICP-OES; Perkin Elmer Life and Analytical, Shelton, USA).

Total carbon was determined by dry combustion (Tiessen et al., 1981) using a Leco TruMac CN Combustion analyser and Inorganic C by Inorganic Carbon Analyser- Skalar Primacs (Skalar Analytical BV, Breda, Netherlands).

Available phosphorus (*P*_Olsen_) was extracted by the sodium bicarbonate method as described by Olsen et al. (1954). Phosphorus in the bicarbonate solution was determined by the phospho‑molybdenum blue method on the Skalar SAN^PLUS^ System (continuous colorimetric flow analysis).

The phosphorus buffer index, a measure of the soil's ability to fix phosphorus, (PBI) was measured with the method of Rayment and Lyons (2011). A single addition of phosphorus (KH_2_PO_4_ in 0.1 M CaCl_2_) at 1000 mg P kg^−1^ was added to the soil at a 1:10 soil to solution ratio. The soil solution was shaken, filtered and then analysed with a Skalar San++ Colorimetric, Continuous Flow Analyser. The PBI index calculation was performed following Rayment and Lyons (2011) using the equation

(1) $$\mathrm{PBI}\;=\;\frac{P_{s}+4.59P_{\text{Olsen}}}{0.41P_{c}},$$

where *P*_s_ is the P sorbed (mg P kg^−1^ soil) and *P*_c_ is the final solution P concentration (mg P L^−1^).

### 2.4 Exhaustive covariates

In addition to the measurements of Se concentration in grain and soil, and associated soil properties, at each sample site, we made use of several environmental covariates, for which values could be extracted at sample sites and which were known at all points on a grid across the study area for spatial mapping. These were the CHELSA downscaled mean annual temperature and precipitation (Karger et al., 2017a, Karger et al., 2017b), the Enhanced Vegetation Index (EVI) derived from the MODIS remote sensor platform (Justice et al., 1997), the original reflectance data from the MODIS satellite in Bands 1, 2, 3 and 7, slope derived from the 30-s resolution MERIT Digital Elevation Model (DEM) of Yamazaki et al. (2017) and topographic index derived from the same DEM, a measure of the tendency for water to accumulate at a site due to surface flow. The values for these covariates were extracted from the grid cells including all the soil–crop sampling sites described in 2.1, 2.2 above.

### 2.5 Statistical analysis

#### 2.5.1 The spatial linear mixed model and the associated spatial predictor

The objective of this analysis is to obtain spatial predictions of the Se concentration in grain in the dominant crops (wheat and teff) across the study region. To do this we use a spatial multivariate linear mixed model (LMM). In this presentation we assume that the target grain for mapping is teff, but the same approach was used to map Se concentration in wheat grain. In the LMM measured Se concentration in teff grain, the concentration in wheat grain and a site-specific prediction of Se concentration in the target grain from measured soil properties are treated as jointly spatially correlated random variables, they are the vectors of variables **y**_1_ (Se concentration in teff grain), **y**_2_ (Se concentration in wheat grain) and **y**_3_ (predicted concentration) in the following expression:

(2) $$\left[\begin{array}{c} y_{1}\\ y_{2}\\ y_{3} \end{array}\right]\;=\;X\left[\begin{array}{c} \;\tau_{1}\\ \;\tau_{2}\\ \;\tau_{3} \end{array}\right]+\left[\begin{array}{c} \eta_{1}\\ \eta_{2}\\ \eta_{3} \end{array}\right]+\left[\begin{array}{c} \;\varepsilon_{1}\\ \;\varepsilon_{2}\\ \;\varepsilon_{3} \end{array}\right].$$

On the right-side of this equation, the matrix **X** contains covariates (the variables referred to in section 2.4), the terms  ***τ***_1_,  **τ**_2_,  ***τ***_3_ are sets of regression coefficients which can be used to predict the expected values of the variables from the covariates. The two remaining sets of terms are random variables, which model the variation in the measured variables unexplained by the covariates. The first set, ***η***_1_, ***η***_2_, ***η***_3_ are spatially correlated random effects, which show spatial dependence and are also mutually correlated (representing, for example, correlation between concentrations of Se in grain of teff and wheat). The second set,  ***ε***_1_,  ***ε***_2_,  ***ε***_3_, are spatially uncorrelated random effects, but may be mutually correlated, representing that variation which occurs at finer spatial scales than is resolved by sampling.

A fuller account of this spatial multivariate LMM is given by Marchant et al. (2009) and by Orton et al. (2014) and more details are in section S.1 of the Supplementary Materials to this paper. Parameters of the model, specifically the variances of the random effects, and parameters which describe the spatial dependence of the spatially correlated random effects, are estimated by maximum likelihood (ML) or residual maximum likelihood (REML) as described in S.1. The fixed effects coefficients can then be estimated by a generalized least-squares procedure. Predictions of the primary variable (e.g. Se concentration at teff grain) can then be computed at unsampled sites where only the values of the covariates are known. These predictions are known as the empirical best linear unbiased predictor (*E*-BLUP), and have an associated prediction error variance (PEV) which quantifies their uncertainty. What makes this approach powerful for our task is that wheat grain Se concentration (when teff grain Se concentration is the target variable for prediction), and the soil observations at sites where no teff was sampled can contribute to the prediction of Se concentration in teff grain by a cokriging process, in so far as the variables are found to be mutually spatially correlated.

#### 2.5.2 Implementing the model

**Fig. 1:**
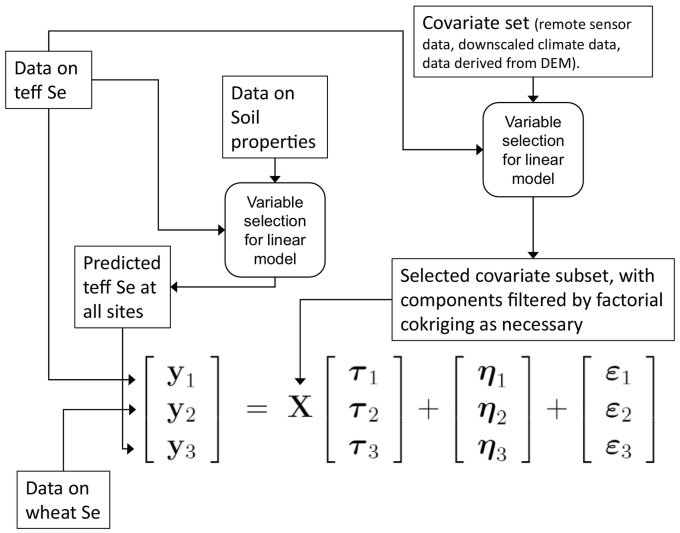
Schematic diagram showing the linear mixed model used in this study and the steps to set up the dependent and independent variables. Note that the model as set out here is for prediction of Se concentration in teff.

The implementation of the spatial LMM is summarized in Fig. 1. It entails a combination of the LMM with a variable selection procedure. This is summarized below.

To make the assumption that the random terms in the LMM were normally distributed, the grain Se concentrations were transformed to natural logarithms. Summary statistics were also computed for the soil properties measured at sample sites (Table 1), and those showing pronounced skewness were also transformed to natural logarithms.

**Table 1:** Summary statistics of soil properties proposed as predictors of grain Se concentration.

Variable	Original units					log⁎-transformed				
	Units	Mean	Median	Standard	Skewness	Mean	Median	Standard	Skewness	Transformed?
				deviation			deviation			
Se_Nit_	μ g kg^−1^	2.54	2.02	1.63	1.86	0.77	0.71	0.55	0.41	Y
Se_Pho_	μ g kg^−1^	5.46	5.11	2.34	1.09	1.61	1.63	0.44	−0.34	Y
Se_TMAH_	μ g kg^−1^	272.86	277.62	145.52	0.30	5.43	5.63	0.66	−0.75	N
pH		6.74	6.84	0.92	−0.14					N
Total oxides	mg kg^−1^	13,469.61	13,149.13	5623.90	0.55	9.42	9.48	0.44	−0.21	N
S_Nit_	mg kg^−1^	37.86	40.14	11.02	−0.62	3.58	3.69	0.36	−1.24	N
S_TMAH_	mg kg^−1^	5.79	4.75	4.04	2.14	1.56	1.56	0.63	−0.24	Y
I_Pho_	μ g kg^−1^	181.53	134.33	132.16	1.15	4.94	4.90	0.75	−0.13	Y
SOC	%	1.46	1.48	0.64	0.46	0.27	0.39	0.51	−0.75	N
Oxalate P	mg kg^−1^	574.50	351.85	609.34	1.97	5.85	5.91	1.14	−0.65	Y
PBI		45.88	41.11	27.49	1.68	3.66	3.72	0.59	−0.25	Y

The subscripts Nit, Pho and TMAH denote the soluble (nitrate extraction), exchangeable (phosphate extraction) and organic (TMAH extraction) fractions in all cases, as described in section 2.3.2. SOC denotes soil organic carbon, and PBI phosphorus buffer index.

⁎ Natural logarithms in all cases.

The first modelling step was to generate the third variable, **y**_3_, in the multivariate set, which is predicted Se concentration in the grain of interest (teff in this example), derived from soil data. This can be computed for every sample site including those where teff was not observed. This prediction was obtained from a linear mixed model in which soil properties were included as fixed effects. To select soil properties for prediction of grain Se concentration we fitted by maximum likelihood (ML) an initial ‘null’ model to sample data in which the only fixed effect for the target grain concentration was a constant mean. We then added soil properties as fixed effects to the model one-by-one, (based on a pre-determined sequence, discussed below) at each step using a log-likelihood ratio test (Section S.2 in the Supplementary Material). If the null hypothesis was rejected then the soil property was retained in the model and the process was repeated, considering the next-listed predictor.

This sequential procedure comprises multiple hypothesis testing which we addressed by controlling the False Discovery Rate (FDR) at 0.05 (Benjamini and Hochberg, 1995). To maintain statistical power, we used the *α*-investment method of Foster and Stine (2008), as implemented by Lark (2017). This requires that the tests are conducted in an a priori order under which the least plausible null hypotheses (i.e. effects thought most likely to be significant) are tested first. This initial process of ordering must be done without reference to the data on grain Se concentration. However, we did examine correlations among the soil properties themselves, because one reason to rank a predictor low in the order is if it is substantially correlated with a predictor already included, and so is unlikely to add much additional information. The ordering was decided through discussion with soil chemists and crop nutritionists on the project team. At this stage we also considered the uncertainty in the determination of soil properties, as judged from detection limits. It should be noted that the validity of the resulting model, and the success of false discovery rate control do not depend on the ordering, which serves simply to improve the probability of detection of a valid predictor given the use of FDR control to avoid over-fitting.

The same variable selection procedure was then used to select the covariates used for prediction, which appear in the matrix **X** in Eq. [2] and Fig. 1. This resulted in a set of candidate environmental covariates for the final LMM. However, it was recognized that such covariates may show spatial variation at nested spatial scales, and it is not necessarily the case that the variation at all scales is predictive of the soil property of interest. For this reason the selected covariates were all subject to factorial kriging analysis (Matheron, 1982) which decomposes a spatial variable into additive components at different spatial scale, see section S.3 in the Supplementary Material. The selected covariates were substituted with their factorial kriging components in the LMM, and those components were retained only if their standardized coefficients fell outside the interval [−2, 2].

#### 2.5.3 Model validation

We used a cross-validation procedure to evaluate the PEVs of the E-BLUP. To do this, the E-BLUP of grain Se concentration at each location, and its PEV, were computed in turn after first deleting the measurement of grain and the predicted grain Se concentration at that site. The cross-validation prediction therefore depended only on the grain Se concentration (both crops) observed at neighbouring sites, and the values of the selected environmental covariates (and factorial kriging components of these) at the sample site. The cross validation procedure was also done using ordinary kriging for prediction from the observations on the Se concentration in grain in the target crop only. The median standardized squared prediction errors were then examined and compared with the 95% confidence interval for the statistic assuming valid PEVs (see supplementary material section S.4; Lark, 2009).

#### 2.5.4 Mapping

The cross-validated models were then used, along with the observed grain Se concentrations, predictions at all sample sites and the environmental covariates to compute the E-BLUP of Se grain concentration for teff and for wheat, separately, on the regular grid of locations at which the environmental covariates were recorded. In addition to the prediction at each location we used the E-BLUP PEV to compute the probability, assuming normal prediction errors, that the grain Se concentration fell below 0.183 mg kg^−1^, the concentration such that a 300-g daily intake of the grain would provide the recommended daily allowance (RDA) for adults of 55 μg day^−1^ Se (Institute of Medicine of the National Academies, 2002).

## 3 Results

### 3.1 Summary statistics, orderings of predictors and variable selection

The basic summary statistics of soil properties (Table 1) showed that several were markedly positively skewed. Those for which the skewness coefficient exceeded 1 were transformed to natural logarithms.

**Fig. 2:**
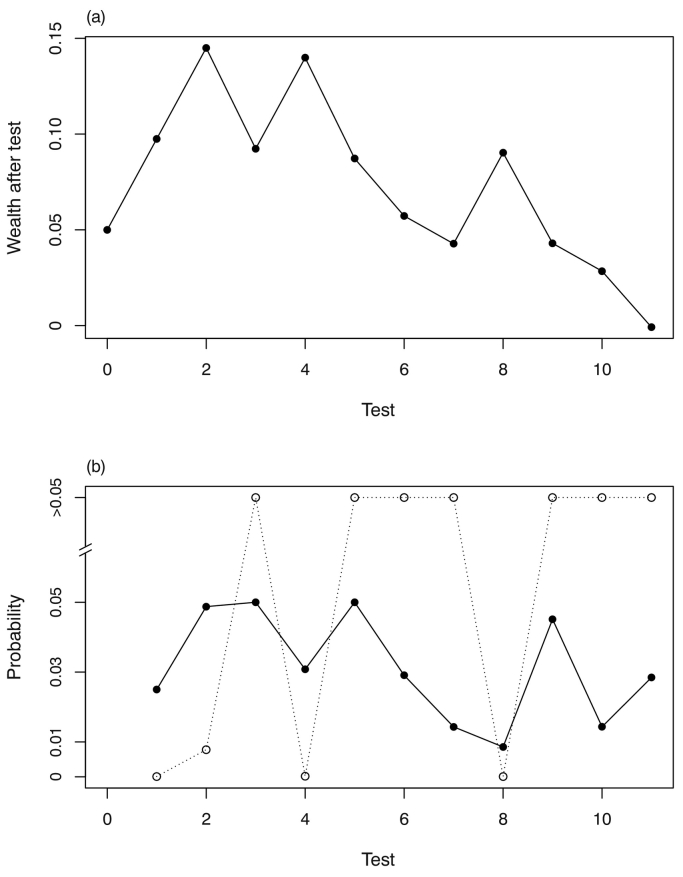
Ordered tests for site (soil) variable selection, teff grain Se. The sequence of predictors is as given in Table 2. The graph at the top (a) shows the *α*-wealth over the sequence of tests and the lower graph (b) shows the *p*-values for successive tests (open symbols) and the corresponding threshold values with marginal false discovery rate control.

The selected order for testing soil properties for prediction of grain Se concentration at a site is shown in Table 2. The rationale for the ordering is summarized in Section S.5 of the Supplementary Material. The process of variable selection for prediction of teff grain Se concentration from soil properties, based upon this ordering, is shown in Fig. 2. The solid symbols in Fig. 2(b) show the threshold *p*-value for the sequential testing procedure for FDR control, and the open symbols show the *p*-values obtained. On this basis we can see that Soluble Se (nitrate extraction), Exchangeable Se (phosphate extraction), soil pH and Exchangeable I (phosphate extraction) were selected as predictors in the case of teff. The comparable plot for wheat grain teff is shown in Fig. S4 in the supplementary material, where Organic Se (TMAH extraction) and soil pH were selected because their *p*-values in the sequential fitting fell below the threshold for FDR control. The parameters for these fitted models, along with the null model in each case (fixed effect a constant mean only) are presented in Table 3.

**Table 2:** Sequence of predictors for grain Se concentration (both soil properties and environmental covariates) for testing with *α*-investment.

Order	Soil Property	Environmental covariate
1	Se_Nit_	Downscaled mean annual precipitation
2	Se_Pho_	Downscaled mean annual temperature
3	Se_TMAH_	Slope
4	pH	Topographic index
5	Sum of oxalate-extractable Fe, Al and Mn oxides	Enhanced vegetation index
6	S_Nit_	MODIS Band 7
7	S_TMAH_	MODIS Band 1
8	I_Pho_	MODIS Band 2
9	SOC	MODIS Band 4
10	Oxalate extractable P	
11	PBI	

The subscripts Nit, Pho and TMAH denote the soluble (nitrate extraction), exchangeable (phosphate extraction) and organic (TMAH extraction) fractions in all cases, as described in section 2.3.2. SOC denotes soil organic carbon, and PBI phosphorus buffer index. Environmental covariates are described in section 2.4.

**Table 3:** Fitted models for soil properties and grain Se concentration in teff and wheat. The symbols used for soil variables are as in Table 1, Table 2.

Predictand		Predictor and coefficient					${\overset{⌣}{R}}_{\mathrm{adj}}^{2}$	*κ*	*τ*^2^	*σ*^2^	*ϕ*
		*β*_0_	*β*_1_	*β*_2_	*β*_3_	*β*_4_					
Teff Se											
	Null model							0.5	0.664	1.052	133.77
			Soil Se_Nit_	Soil Se_Pho_	pH	Soil I_Pho_					
		−2.820	0.924	−0.221	0.316	−0.496	0.58	0.5	0.500	0.223	30.63
Wheat Se											
	Null model							0.5	0.562	0.816	16.00
			Soil Se_TMAH_	pH							
		−6.66	−0.001	0.546			0.27	0.5	0.596	0.407	11.53

**Fig. 3:**
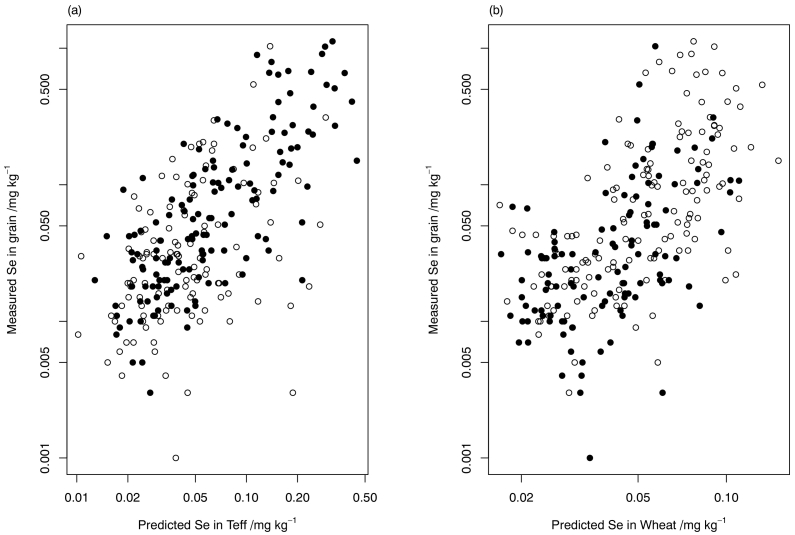
Plot of grain Se concentration predicted with a model for (a) teff and (b) wheat for all observations at all sites, against the measured grain Se concentration at the site. In each plot a solid symbol represents a site where the observed grain concentration is for the same crop species for which the model was fitted – e.g. an observed teff grain Se concentration and predicted teff grain Se concentration at the site in (a); and an open symbol represents a site where the observed grain Se concentration is for the species other than the one for which the mode was fitted – e.g. an observed wheat grain Sec concentration and the predicted teff grain concentration for that site in (a).

The predicted concentrations of Se in teff grain were then computed for each site from the soil information. They are plotted in Fig. 3 (a) against the measured Se concentration in grain at each site. The solid symbols correspond to sites where the observed grain was teff, and so these points give a visual impression of the goodness of fit of the model fitted with FDR control. The open symbols correspond to the sites where the observed grain was wheat. Conversely Fig. 3 (b) shows a plot of the predicted Se concentrations in wheat grain at each site against the observed Se concentration in grain at each site, with solid symbols at sites where the observed grain was wheat, and open symbols where it was teff.

For those sites where wheat was grown, the predicted Se concentration for teff grain with the same model is plotted against the observed wheat Se concentration. The comparable plot for predicted Se concentration in wheat grain is shown in Fig. 3 (b), where the solid symbols are used for the observed concentrations in wheat grain and the open symbols.

The selected order of environmental covariates for spatial prediction of Se concentration in grain is shown in Table 2. The rationale for this ordering is summarized in section S.6 of the Supplementary Material.

**Fig. 4:**
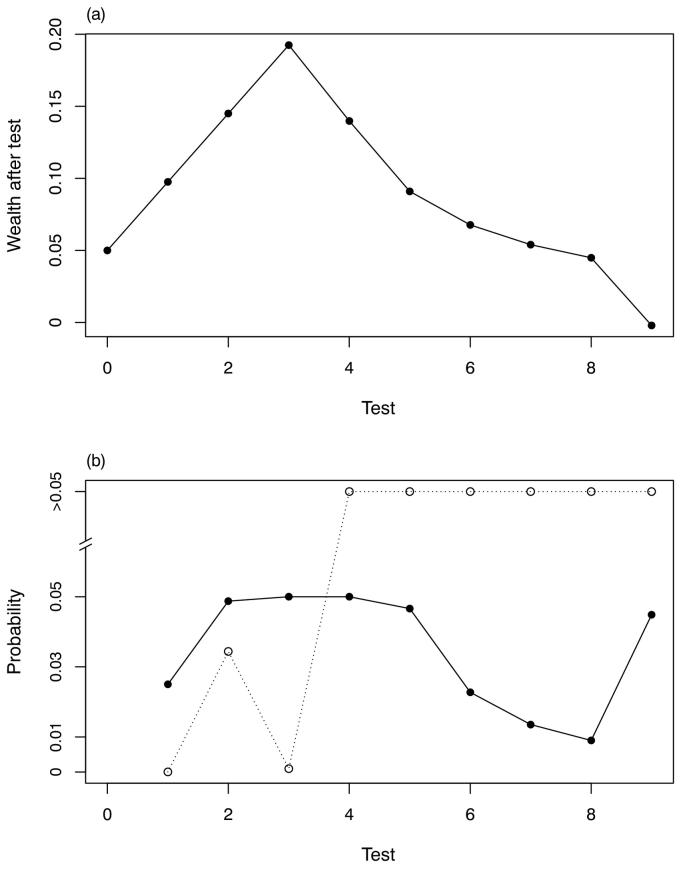
Ordered tests for covariate selection, teff Se. The sequence of predictors is as given in Table 2. The graph at the top (a) shows the *α*-wealth over the sequence of tests and the lower graph (b) shows the *p*-values for successive tests (open symbols) and the corresponding threshold values with marginal false discovery rate control.

Fig. 4 shows the output of the sequential testing of predictors for teff grain Se concentration from among the environmental covariates. Downscaled mean annual precipitation and temperature and slope, the first three covariates in the sequence, were selected because their *p*-values were below the threshold for FDR control with *α*-investment. The comparable results for wheat grain are shown in Fig. S5 in the supplementary material. The plot shows that the *p*-values for none of the covariates was smaller than the corresponding threshold, so none were selected. The model parameters for both grains are presented in Table 4.

**Table 4:** Fitted models for covariates and grain Se concentration in teff and wheat. The covariates are described in section 2.4. NB The model presented for wheat was not selected under the control of marginal false discovery rate, but is included for comparison.

Predictand		Predictor and coefficient					${\overset{⌣}{R}}_{\mathrm{adj}}^{2}$	*κ*	*τ*^2^	*σ*^2^	*ϕ*
		*β*_0_	*β*_1_	*β*_2_	*β*_3_	*β*_4_					
Teff Se											
	Null model							0.5	0.664	1.052	133.77
			Precipitation	Mean annual	Slope						
				temperature							
		−4.227	−0.001	0.016	0.073		0.49	0.5	0.568	0.310	47.02
Wheat Se											
	Null model							0.5	0.562	0.816	16.00
			EVI	MODIS Band 7							
		−0.435	−0.0005	−0.0014			0.18	0.5	0.559	0.565	14.76

### 3.2 Linear mixed model fitting and cross-validation

**Fig. 5:**
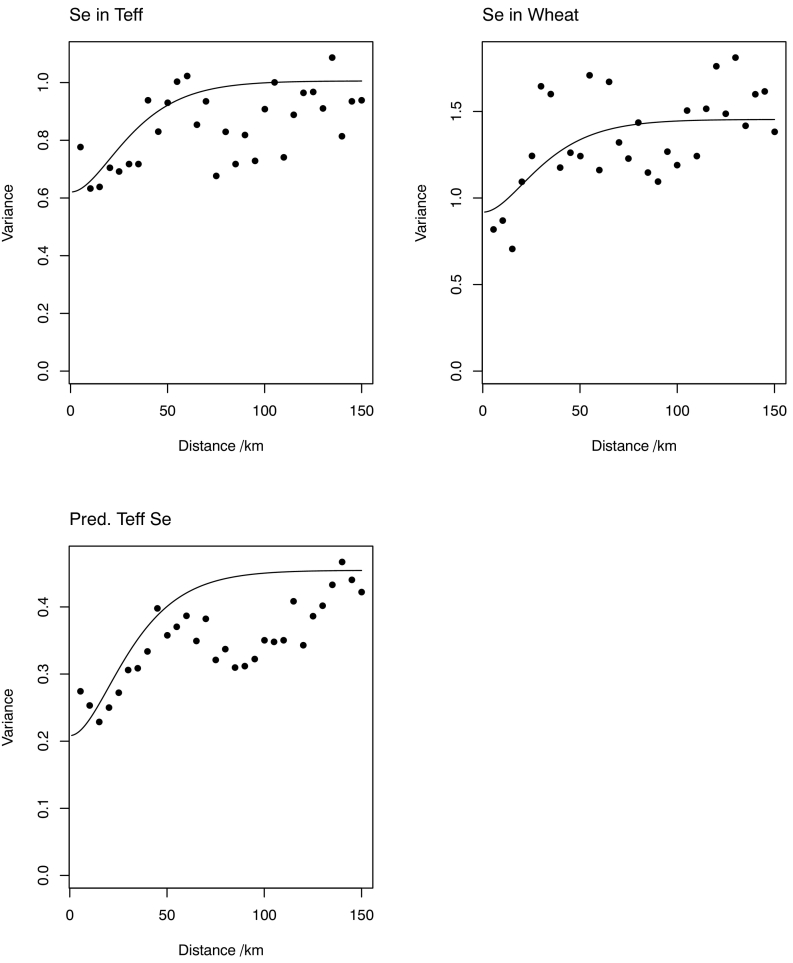
Autovariograms from teff Se LMCR.

For the LMM to predict teff Se concentration, the smoothness parameter, *κ*, of the spatially correlated random effects was set at a value of 2.0 based on the profile likelihood for a model with all predictors included (Fig. S6 in the supplementary material). The environmental covariates, slope and mean annual temperature were decomposed into short-range and long-range components by factorial kriging. In the initial fitting of the model the standardized coefficient for the short-range components of these variables were small, and they were dropped, indicating that the evidence for a relationship between these variables and teff grain Se concentration at the variable selection stage arose from the long-range variability of these variables. The fitted model parameters for the final LMM for each variable, including the correlation matrices for the random components, are shown in Table 5(a,b). These tables include the correlations between the random components in the respective models, both the spatially correlated random effects (***η***_1_,***η***_2_,***η***_3_) and the uncorrelated or ‘nugget’ components,(***ε***_1_, ***ε***_2_, ***ε***_3_). Note that, for the spatially correlated components, there are moderate correlations between the random effects for wheat and teff Se, and between the observed grain Se concentration and that predicted from soil properties. Fig. 5 shows the empirical variograms for the marginal residuals of Se concentration in teff and wheat grain and the predicted concentration in teff grain from soil data (all on a log-scale) in the fitted LMM, with the corresponding variogram models from the parameters of the LMM. Note that the models are not fitted to the empirical variograms as such, and that differences are expected due to both the bias in the empirical variogram in the presence of a non-constant fixed effect (Cressie, 1993) and the constraints of the multivariate LMM (Webster and Oliver, 2007). In the case of the LMM for prediction of wheat grain Se concentration a smaller value of *κ*, 0.5, was selected (Fig. S7 in the supplementary material), the empirical variogram and fitted LMCR for the random component of the model are shown in Fig. S8 in the supplementary material.

**Table 5:** Linear mixed model parameters.

(a) With selected covariates for Se concentration in teff grain.			
Dependent variable	Fixed effect	Coefficient	Standard error
*Fixed effects parameters*			
Teff Se	Constant	1.64	0.94
	Precipitation (long-range)	−0.0037	0.0008
Wheat Se	Constant	−3.54	0.23
Predicted teff Se	Constant	−2.61	0.94
	Temperature	0.0088	0.0026
	Precipitation	−0.0014	0.0005
	(long-range)		

*Random effects parameters*			
	*κ*	2.0	
	*ϕ*	14.92	
Nugget variances	Teff Se	0.62	
	Wheat Se	0.92	
	Predicted Teff Se	0.21	
Correlated variances	Teff Se	0.39	
	Wheat Se	0.54	
	Predicted teff Se	0.25	


Correlation matrices (linear model of coregionalization with grain Se concentration and predicted teff Se concentration)			
	Teff Se	Wheat Se	Predicted Teff Se
Nugget			
Teff Se	1.00		
Wheat Se	0.00	1.00	
Predicted teff Se	0.43	0.20	1.00

Spatially correlated			
Teff Se	1.00		
Wheat Se	0.44	1.00	
Predicted teff Se	0.52	0.42	1.00


(b) With selected covariates for Se concentration in teff grain.			
Dependent variable	Fixed effect	Coefficient	Standard error Teff Se
*Fixed effects parameters*			
Teff Se	Constant	−2.66	0.29
Wheat Se	Constant	−3.14	0.34
Predicted wheat Se	Constant	−3.26	0.17

*Random effects parameters*			
	*κ*	0.5	
	*ϕ*	49.29	
Nugget variances	Teff Se	0.63	
	Wheat Se	0.73	
	Predicted wheat Se	0.08	
Correlated variances	Teff Se	0.69	
	Wheat Se	0.89	
	Predicted wheat Se	0.27	


Correlation matrices (linear model of coregionalization with grain Se concentration and predicted wheat Se concentration)			
	Teff Se	Wheat Se	Predicted Wheat Se
Nugget			
Teff Se	1.00		
Wheat Se	0.00	1.00	
Predicted wheat Se	0.19	0.14	1.00

Spatially correlated			
Teff Se	1.00		
Wheat Se	0.64	1.00	
Predicted wheat Se	0.53	0.80	1.00

**Fig. 6:**
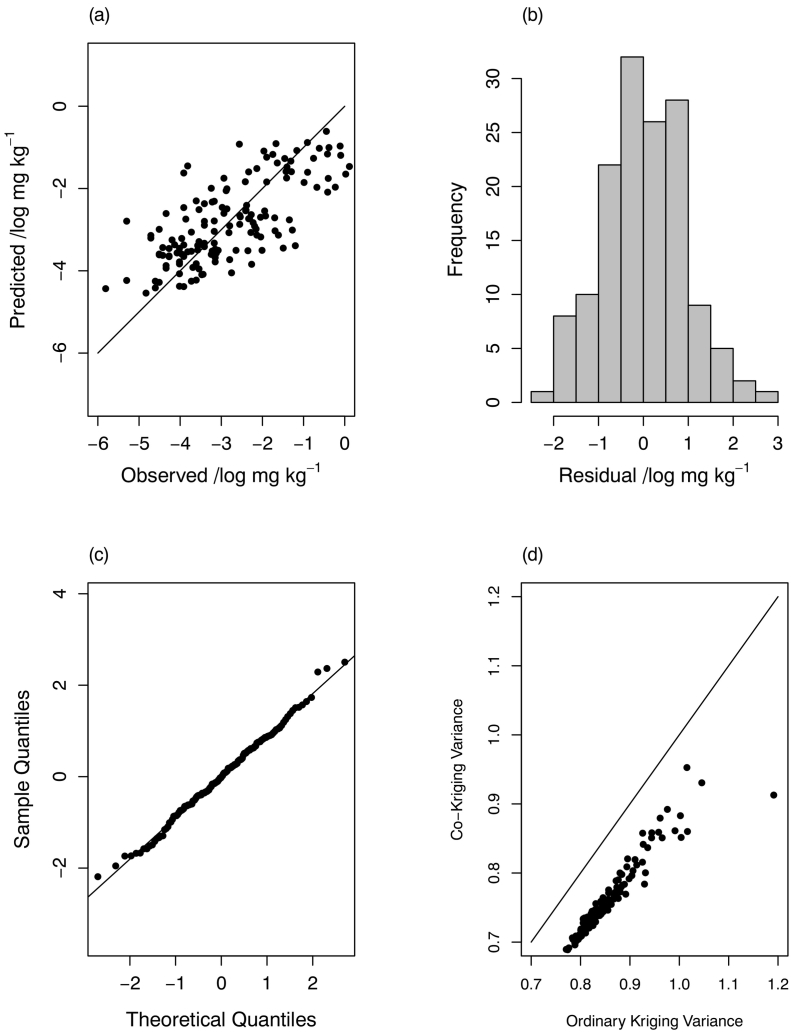
Cross-validation plots (CoK) for teff Se LMCR.

**Fig. 7:**
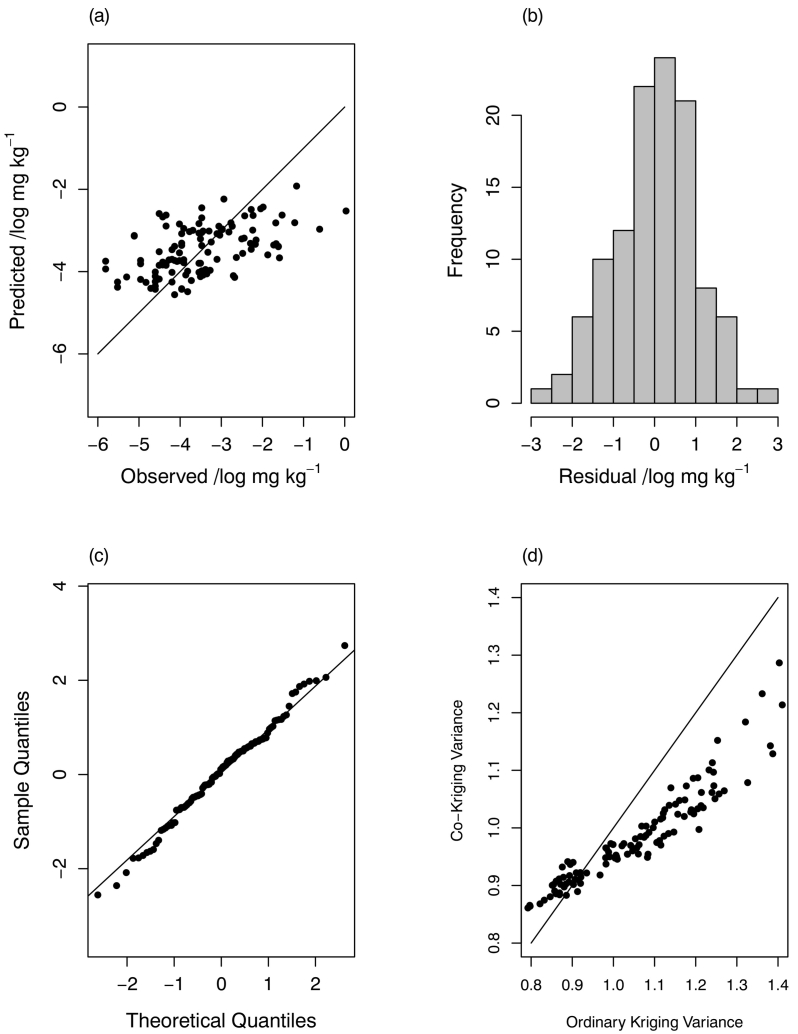
Cross-validation plots (CoK) for wheat Se LMCR.

The distributions of cross-validation errors for prediction of grain Se by ordinary kriging are shown in Figs. S9 and S10 of the supplementary material. The cross-validation errors for the E-BLUP from the multivariate LMM results are shown in Fig. 6, Fig. 7. The assumption of normal prediction errors appears to be plausible, and the summaries of the standardized squared prediction errors in Table 6, support the validity of the models. The plots of the prediction error variances for the two predictions (Fig. 6(d) and 7(d)) show the advantages of incorporating the covariates and the coregionalized variables into the model through the reduction of the kriging variance.

**Table 6:** Cross-validation results for final predictive models, and ordinary kriging for comparison.

Predictand	Predictor		Mean SSPE^a^	Median SSPE^b^
Teff Se	OK^c^		1.00	0.35
	LMCR–		1.09	0.47
	E-BLUP^d^			
Wheat Se	OK		1.01	0.41
	LMCR–		1.00	0.36
	E-BLUP			

a Standardized square prediction error.

b The 95% confidence interval for the Teff set is {0.28,0.63} and for the wheat set it is {0.26,0.65}.

c The ordinary kriging predictor.

d The empirical best linear unbiased predictor conditional on the multivariate linear mixed model.

### 3.3 Spatial predictions of Se concentration in grain

**Fig. 8:**
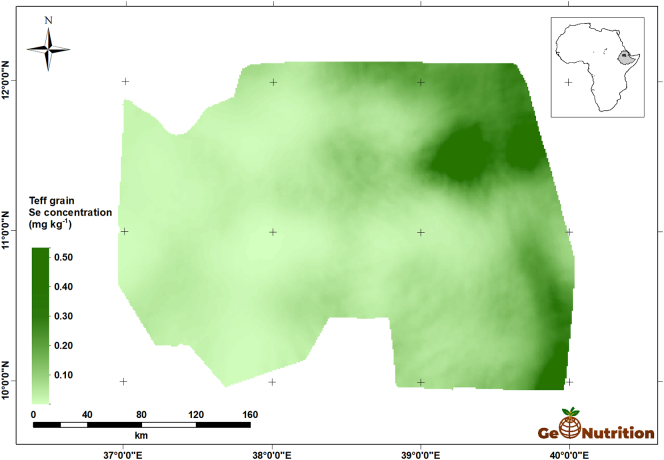
Median unbiased prediction of Se concentration in teff grain across the study region.

**Fig. 9:**
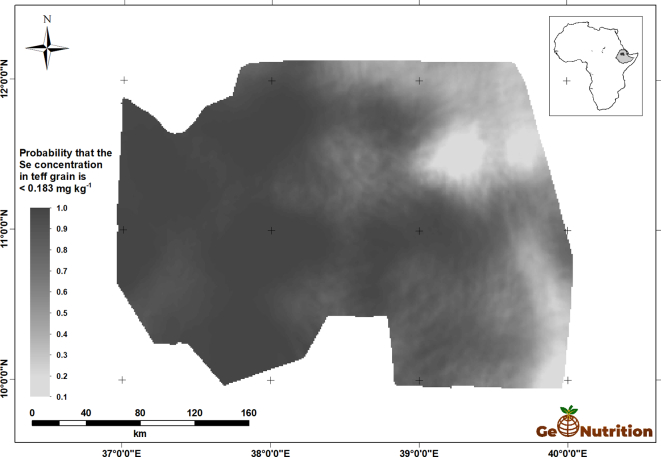
Probability that Se concentration in wheat grain <0.183 mg kg^−1^.

**Fig. 10:**
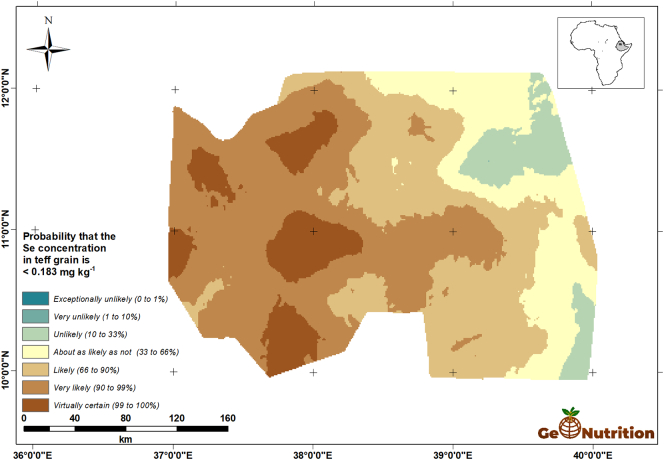
Probability that Se concentration in teff grain <0.183 mg kg^−1^ using a verbal scale of calibrated phrases from Mastrandrea et al. (2010).

**Fig. 11:**
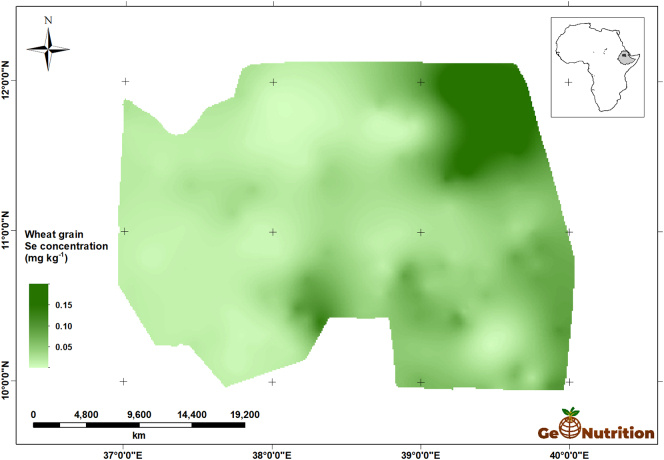
Median unbiased prediction of Se concentration in wheat grain across the study region.

**Fig. 12:**
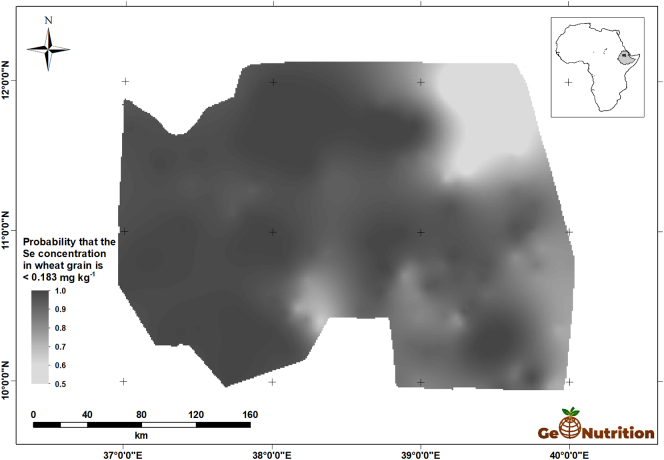
Probability that Se concentration in wheat grain <0.183 mg kg^−1^.

**Fig. 13:**
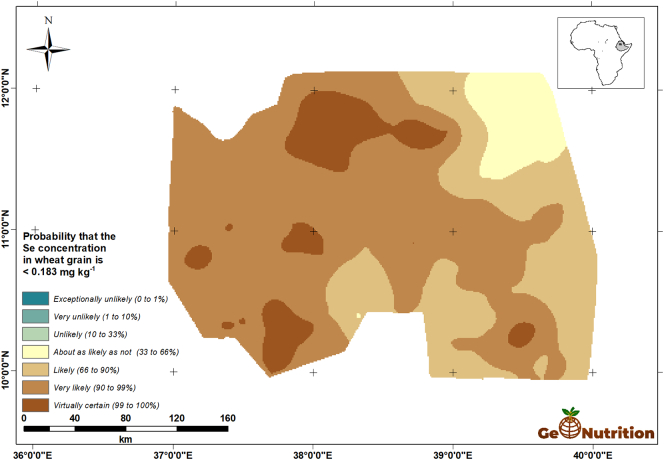
Probability that Se concentration in wheat grain <0.183 mg kg^−1^ using a verbal scale of calibrated phrases from Mastrandrea et al. (2010).

Fig. 8 shows the spatial predictions of teff Se concentration across the study area. There are clear trends, with larger concentrations in general in the east of the region, and some marked variations over shorter distances, consistent with the variograms in Fig. 5. Fig. 9 shows that, over most of the region, the probability that grain Se concentration is insufficient to provide the RDA of Se from a 300-g intake is large. The interpretation of these probabilities is facilitated by representing them on a scale which represents the probability in terms of the calibrated verbal phrases of Mastrandrea et al. (2010) in Fig. 10. The maps for wheat Se concentration (Fig. 11, Fig. 12, Fig. 13) show comparable spatial patterns, which is not surprising, given the moderate correlation between the spatially correlated random effects for Se concentration in the two grains reported in Table 5(a) and (b).

## 4 Discussion and conclusions

This study shows how joint sampling of soil and grain, with an appropriate sampling design and model-based statistical analysis, allows us to examine the spatial variation of cereal composition with respect to micronutrient concentration over large regions and to represent it as a map. We are not aware of any previous study that has shown the spatial variation of a key determinant of a population's micronutrient supply with comparable spatial resolution.

It appears that the risk of Se deficiency, resulting from a diet in which wheat or teff is a staple, is largest in the west of Amhara Region. There are differences between the crops, however, with wheat less likely to provide sufficient Se intake than teff across the whole area. In small parts of the east of the study area it is judged ‘unlikely’ that a 300-g daily intake of teff would fail to provide the RDA of Se. Such spatial information on potential intake of Se from staple crops could clearly be used to improve the targeting of interventions to address deficiency.

A map of Se concentration in grain is of greater use for the identification of regions at risk of deficiency than maps of soil properties alone, as no assumptions must be made about soil-to-crop transfer. That said, our approach made use of soil measurements at sites collocated with the target grain samples, as well as at sampling sites where other crops were grown, by integrating predicted grain Se concentration from these data into the multivariate LMM. The predictive models for Se concentration were based on soil properties selected with false discovery rate control to avoid over-fitting, and so they merit examination. Soil pH was a selected predictor for Se concentration in both teff and wheat grain, with a positive coefficient implying that, other factors remaining constant, less Se is expected in grain over more acid soils. This is consistent with results found in Malawi (Chilimba et al., 2011) and elsewhere in Africa (Ligowe et al., 2020). The interpretation of linear models and their coefficients must always be cautious because of correlations among the predictors, but on the basis of this result it would be worth examining whether liming the more acid soils would improve grain Se concentration. It should be noted, though, that these soils are not particularly acid. The median pH is 6.84, the first quartile is 5.98 and 90% of the samples had a pH in excess of 5.49. Similarly, extractable Se in the soil appeared in both predictive models, although Se obtained with different extractants were selected for prediction of Se in wheat and teff grain. This suggests that a soil test could be developed to make site-specific predictions of Se concentration in grain.

Despite the use of methods for variable selection that avoid over-fitting, and the fact that our predictions are optimal in the sense of being the best linear unbiased prediction, there is inevitable residual uncertainty in the predictions. Our cross-validation procedure suggests that this uncertainty is well-characterized by the prediction error variance supplied by the model, and so we can quantify the residual uncertainty. In this study we used established methods to represent this uncertainty in the spatial predictions — while a data-user interested in a particular location can obtain a prediction of Se concentration in grain there, they can also obtain the probability that the true value falls below a threshold of interest to nutritionists, and this can be expressed on a verbal scale which may facilitate communication to a wider audience.

This study has demonstrated some innovative approaches to spatial modelling for prediction. First, by using false-discovery rate control with *α*-investment we were able to select variables for Se prediction from soil properties with confidence that we are not over-fitting, while at the same time maintaining statistical power by testing hypotheses in a sequence determined by prior knowledge and informed hypothesizing about underlying processes. The fitted model may therefore merit further examination for insight into soil factors influencing grain Se concentration, as noted above. One should bear in mind, of course, that the failure to select a variable does not necessarily mean that it has no bearing on the process of interest. One underlying reason that a variable might be rejected is because it is strongly correlated with one already in the model, or because it is measured with substantial error.

Second, we can be confident that we are not over-fitting covariates, and indeed none were selected for the prediction of Se concentration in wheat grain. By filtering covariates, where appropriate, by factorial kriging, and testing the predictive value of the different components separately, we also avoided introducing spurious short-range variation into our predictions. It would clearly be wrong, for example, to allow short-range variation in down-scaled precipitation to induce comparable variation in predicted grain Se concentration when the two variables are related because of regional-scale climatic covariation. We avoided this by factorial kriging analysis (FKA). There was no evidence that the short-range component of this covariate extracted by FKA was related to grain Se concentration, and only the long-range component was included in the predictive model.

Finally, our multivariate LMM had smaller prediction error variances than did ordinary kriging (Fig. 6(d) and 7(d)). This improved prediction can be attributed to the covariates used in the model, and to the cross-coregionalization with the site information and grain Se concentration at sites where the non-target crop was grown. In this way the multivariate LMM allows us to make maximum predictive use of relationships among variables measured in the field sampling, even when these are not collocated with the particular target variable of interest.

There is scope for further development of the work reported in this paper. First, the sampling and statistical methodology can be extended to other mineral micronutrients that may be deficient both in this region and elsewhere. Second, there is potential to combine the predicted concentrations of micronutrients in grain with food consumption data to improve estimates of dietary mineral intakes and, potentially, to target future investments to alleviate deficiencies. Finally, one might compare these inferences about spatial variation in intake with spatial data on human biomarkers for nutrient deficiency to validate the implict hypothesis that spatial variations in staple food micronutrient concentrations will, via intake, induce comparable spatial variations in micronutrient status. Again, this information could help policy makers identify and target efficient interventions.

To conclude, joint sampling of the crop and soil in an appropriate design allowed us to map the spatial variation of grain Se concentration across a large region of Ethiopia, making use of both site-specific soil and grain observations and exhaustive covariates derived from remote sensor data and a digital elevation model. A cross-validation procedure showed that the best linear unbiased predictor and its prediction error variance gave predictions with robust characterization of their uncertainty, and this allowed us to quantify and communicate uncertainty in terms of predicted grain Se concentration and the concentration required to provide the RDA from a standard serving of grain. There is substantial spatial variability in the supply of Se from staple cereal crops, which could be relevant to the design of efficient interventions.

## CRediT authorship contribution statement

**D. Gashu:** Conceptualization, Methodology, Investigation, Writing - original draft, Writing - review & editing, Supervision, Project administration, Funding acquisition. **R.M. Lark:** Conceptualization, Methodology, Software, Validation, Formal analysis, Writing - original draft, Writing - review & editing, Visualization, Funding acquisition. **A.E. Milne:** Conceptualization, Methodology, Software, Validation, Formal analysis, Writing - original draft, Writing - review & editing, Funding acquisition. **T. Amede:** Conceptualization, Methodology, Writing - review & editing, Funding acquisition. **E.H. Bailey:** Methodology, Validation, Investigation, Resources, Writing - review & editing. **C. Chagumaira:** Software, Writing - review & editing, Visualization. **S.J. Dunham:** Methodology, Validation, Investigation, Resources, Writing - review & editing. **S. Gameda:** Conceptualization, Methodology, Writing - review & editing, Funding acquisition. **D.B. Kumssa:** Investigation, Methodology, Writing - review & editing, Data curation. **A.W. Mossa:** Methodology, Validation, Investigation, Resources, Writing - review & editing. **M.G. Walsh:** Methodology, Software, Validation, Investigation, Resources, Writing - review & editing. **L. Wilson:** Methodology, Validation, Investigation, Resources, Writing - review & editing. **S.D. Young:** Methodology, Validation, Investigation, Resources, Writing - review & editing. **E.L. Ander:** Conceptualization, Methodology, Resources, Writing - review & editing, Supervision, Funding acquisition. **M.R. Broadley:** Conceptualization, Methodology, Resources, Writing - review & editing, Supervision, Project administration, Funding acquisition. **E.J.M. Joy:** Conceptualization, Methodology, Investigation, Resources, Writing - review & editing, Supervision, Funding acquisition. **S.P. McGrath:** Conceptualization, Methodology, Resources, Writing - review & editing, Supervision, Project administration, Funding acquisition.

## Appendix A Supplementary data

Supplementary material Image 1
