# Supplementary material for: Spatial prediction of the concentration of selenium (Se) in grain across part of Amhara Region, Ethiopia
Source: Sci Total Environ. 2020 Sep 1;733:139231. doi: 10.1016/j.scitotenv.2020.139231 (PMC7298608; doi:10.1016/j.scitotenv.2020.139231)
Supplement: Supplementary file 1 — Supplementary material [file mmc1.pdf]

Spatial prediction of the concentration of selenium (Se) in grain across part of Amhara Region, Ethiopia

Supplementary Material

S.1 The Multivariate Spatial Linear Mixed Model

Spatial prediction was by the empirical best linear unbiased predictor (E-BLUP) (Stein, 1999) based on a multivariate LMM of the primary variable (Se concentration in a particular grain) and secondary variables (including Se concentration in the non-target grain). In the LMM the joint mean of the primary variable and the secondary variables is modelled in terms of fixed effects (which here can include the environmental covariates), and random effects which are assumed to have a multivariate normal distribution described by the linear model of coregionalization (Journel and Huijbregts, 1978). Details of the multivariate LMM (Marchant *et al.,* 2009; Orton et al., 2014) are summarized below.

If we have a case where two or more variables are to be modelled jointly (e.g. Se concentration in teff grain and of wheat grain, and other spatial variables) then we treat these as a multivariate normal random variable (perhaps after appropriate data transformation). In this case a set of observations of the primary variable, $\mathbf{y}_1$, and the $(m-1)$ secondary variables, $\mathbf{y}_2^{\mathrm{T}}, \cdots, \mathbf{y}_m^{\mathrm{T}}$ observed at $n_1, n_2, \cdots n_m$ locations respectively, is modelled as a length $n = n_1 + n_2 + \cdots + n_m$ random variate:

$$\begin{bmatrix} \mathbf{y}_1 \\ \mathbf{y}_2 \\ \vdots \\ \mathbf{y}_m \end{bmatrix} = \mathbf{X} \begin{bmatrix} \boldsymbol{\tau}_1 \\ \boldsymbol{\tau}_2 \\ \vdots \\ \boldsymbol{\tau}_m \end{bmatrix} + \begin{bmatrix} \boldsymbol{\eta}_1 \\ \boldsymbol{\eta}_2 \\ \vdots \\ \boldsymbol{\eta}_m \end{bmatrix} + \begin{bmatrix} \boldsymbol{\varepsilon}_1 \\ \boldsymbol{\varepsilon}_2 \\ \vdots \\ \boldsymbol{\varepsilon}_m \end{bmatrix}. \quad \text{(S.1)}$$

There are three terms on the right of this equation. The first deals with the fixed effects (environmental covariates in this study). The term $\mathbf{X}$ is a design matrix. This has n rows and $p = p_1 + p_2 + \cdots + p_m$ columns where p_i is equal to the number of fixed effects terms in the model for the ith random variate. In a simple example, if all the variates have one single fixed effect predictor variable in the model then $p_1 = p_2 = \cdots = p_m = 2$, and if the jth element in the vector y corresponds to an observation of the ith variate then $\mathbf{X}[j, 2i]$

is equal to the corresponding value of the predictor variable, $\mathbf{X}[j,(2i-1)]$ is equal to 1, and all other elements in the jth row of $\mathbf{X}$ are zero. The vector $\boldsymbol{\tau}_i$ contains the fixed effects coefficients for the prediction of the ith variate. In this simple example the two terms in $\boldsymbol{\tau}_i$ are the intercept and coefficient of a linear regression of $\mathbf{y}_i$ on the predictor variable in $\mathbf{X}$. The terms $\boldsymbol{\eta}_1, \boldsymbol{\eta}_2, \cdots, \boldsymbol{\eta}_m$ are spatially correlated normal random variables of mean zero, which we assume conform to a LMCR. Let some element of $\boldsymbol{\eta}_i$, where $i \in \{1, m\}$ be denoted by $\eta_i(\mathbf{s})$ where $\mathbf{s}$ is a vector with the coordinates of the observation in space. Under the LMCR the covariance of any two observations separated spatially by a lag vector $\mathbf{h}$: $\eta_i(\mathbf{s}), \eta_i(\mathbf{s}+\mathbf{h})$, is assumed to depend only on the lag vector and is given by

$$K_{i,j}(\mathbf{h}) = \sum_{k=1}^{s} c_k^{i,j} \rho_k(\mathbf{h}|\phi_k, \kappa_k), \tag{S.2}$$

where there are $s \geq 1$ independent additive components in the model and the terms $c_k^{i,j}$ are variances and covariances. The function $\rho_k(\mathbf{h}|\phi_k, \kappa_k)$ is a spatial correlation function which gives the correlation for the kth component of the LMCR over lag $\mathbf{h}$. In this study we assume that the correlation function depends on the lag distance, $|\mathbf{h}|$, not the direction (i.e. that it is isotropic), and can be described by the function due to Matérn (Stein, 1999):

$$\rho(\mathbf{h}|\phi, \kappa) = \frac{1}{2^{\kappa-1}\Gamma(\kappa)} \left(\frac{2\kappa^{\frac{1}{2}}|\mathbf{h}|}{\phi}\right)^{\kappa} \mathcal{K}_\kappa \left(\frac{2\kappa^{\frac{1}{2}}|\mathbf{h}|}{\phi}\right), \tag{S.3}$$

where ϕ is a distance parameter, κ is a smoothness parameter and $\mathcal{K}_\kappa$ is a modified Bessel function of the second kind of order κ.

Each term $\boldsymbol{\varepsilon}_1, \boldsymbol{\varepsilon}_2, \cdots, \boldsymbol{\varepsilon}_m$ is a zero-mean, independently and identically distributed random variable. Under the assumption of normality, these terms are entirely characterized by their variances and covariance, which we denote $c_0^{i,k}$, $i, k \in \{1, m\}$. These terms are spatially uncorrelated, and so they represent components of the variation of our variables which are either not spatially-dependent, or which are spatially dependent at scales too fine to be resolved by the sampling of the variables.

The variances and covariances of the two random terms, and the parameters of the Matérn correlation function may be estimated from observations by maximum likelihood (ML) or by residual maximum likelihood (REML),

as described by Marchant and Lark (2007). These estimates of the random effects parameters can then be used to obtain estimates of the fixed effects coefficients, $\boldsymbol{\tau}_i,\ i \in \{1, m\}$. The REML estimation method is preferred, because it reduces bias in random effects parameters due to the uncertainty in the fixed effects parameters. However, the ML method was used for modelling steps when variable selection was done (see section S.2 below) as the maximized likelihood may be compared between models with different fixed effects structures, but such a comparison is not valid for maximized residual likelihood. For this reason ML was used for all model and variable selection steps, then the final set of model parameters for predictive use were estimated for the selected fixed effects structure by REML. Note that we followed Diggle and Ribeiro (2006) in using a profiling method to obtain an estimate of the κ parameter in Eq (S.3), finding the ML estimate for all other parameters while holding κ fixed at a sequence of values, then selecting the set of estimates for which the maximized likelihood was largest.

Once the fixed and random effects parameters of the LMM have been estimated the model can then be used to obtain predictions of the variables $Y_1, Y_2, \cdots, Y_m$ at sites where they have not been measured but where any predictor variables in X are known. This prediction has two components, the first of which (a regression-type prediction) depends on the value of the covariates and the fixed effects parameters $\boldsymbol{\tau}_i,\ i \in \{1, m\}$. The second component is a spatial interpolation of the random component of the model for the target variable, it is essentially a cokriging prediction, and so is based on estimates of the random effects $\boldsymbol{\eta}_i,\ i \in \{1, m\}$, at the sample sites. The overall prediction, the E-BLUP, has errors, the variance of which can be computed from the LMM. The E-BLUP is the best predictor in the sense that this variance is minimized.

Note that all spatial modelling was done after conversion of the coordinates of all observations to the Universal Transverse Mercator projection zone 37N. The spatial predictions were then transformed back to geographical coordinates (latitude and longitude) for presentation as maps.

S.2 The log-likelihood ratio test

Consider a case where the maximized log-likelihood (not the residual

log-likelihood) for a linear mixed model that we call the null model is ℓ_{N}, and that for a proposed model in which we have added an additional 'candidate' predictor in the fixed effects to the set used in the null model is ℓ_1. At the start of a sequential process of variable selection, as used in this study, the null model is one with a constant mean as the only fixed effect, and the proposed model adds the first-listed predictor variable of interest. One may compare the two models with the log-likelihood ratio statistic:

$$L \;=\; 2\left(\ell_1 - \ell_{\mathrm{N}}\right). \tag{S.4}$$

The statistic L is a measure of the evidence that the added predictor has improved the fit of the model. Under a null hypothesis that there is no relation between the grain Se concentration and the added predictor the distribution of L is chi-square, with degrees of freedom equal to the difference in the number of parameters between the two models, 1 in the case where we have added a single candidate predictor (Verbeke and Molenberghs, 2000). This holds for the regular case where (i) the null model can be regarded as a special case of the proposed model, with the fixed effect coefficient for the candidate predictor set to zero and (ii) the fixed effect coefficient is not bounded at zero. Both conditions hold for all applications in this paper.

S.3 Factorial kriging

When a spatial variable is modelled as the additive combination of two or more spatially correlated random variables, with a combined variogram which is the sum of the variograms for these variables, then factorial kriging is a method which allows one to decompose the data into estimates of the two components (and an uncorrelated nugget component).

The empirical variogram of each covariate was estimated, and nested models were fitted with combinations of up to two spherical and exponential variogram functions. For cases where two nested components were identified (in addition to a spatially uncorrelated nugget effect), the nugget and short-range components of the variable were estimated by factorial kriging, following the account of the method given by Webster and Oliver (2007), which was implemented for the R platform. The long-range component of the variable was then obtained by difference from the original data. The separate factorial kriging estimates of the components of the covariate were then examined as

fixed effects in the final LMM.

S.4 The standardized squared prediction error of cross-validation

The standardized squared prediction error at any sample site at location $\mathbf{s}$ was computed as

$$\theta(\mathbf{s}) = \frac{\left\{ y_i(\mathbf{s}) - \tilde{Y}_i(\mathbf{s}) \right\}^2}{\sigma^2_{\mathrm{BLUP}}(\mathbf{s})}, \tag{S.5}$$

where $y_i(\mathbf{s})$ and $Y_i(\mathbf{s})$ denote, respectively, the observed selenium concentration in the grain sample at location $\mathbf{s}$ and its E-BLUP prediction, and $\sigma^2_{\mathrm{BLUP}}(\mathbf{s})$ denotes the prediction error variance of the E-BLUP, which depends only on the parameters of the LMM used for prediction, the spatial distribution of the neighbouring sample points and the value of the environmental covariates at $\mathbf{s}$. The expected value of the standardized squared prediction error is one, for a valid model, and the most sensitive diagnostic is the median value, with an expectation of 0.455 for normally distributed prediction errors (Lark, 2009).

S.5 Ordering of soil properties as potential predictors of Se concentration in grain

The selected order for testing soil properties for prediction of grain Se concentration at a site is shown in Table 2 of the paper. The rationale for the ordering is summarized below.

1. It was decided that extractable soil Se would be the most likely predictor of grain Se concentration, among soil properties. There was more than one measure of extractable Se in the data set, and all were included at the top of the list in order:

   i. soluble Se (nitrate)

   ii. exchangeable Se (phosphate)

   iii. organic Se (TMAH)

2. Following extractable soil Se, we considered soil properties expected to affect Se mobility in soil in order:

   iv. pH

   v. sum of oxalate-extractable Fe, Al and Mn (metal oxides)

3. We then included soil sulphur concentration, as this may be a competitor with Se for transporters into the plant. We excluded exchangeable sulphur for which 38% of observations were below detection limit, and many were negative, and included the others in the following order:

   vi. soluble S (nitrate)

   vii. organic S (TMAH)

4. We then included exchangeable iodine which could be a proxy of Se content of soil originating in rainfall. Because this is a proxy effect, and the extractable Se is already included, it was given a relatively low rank.

   viii. exchangeable I (phosphate)

5. It was suggested that soil organic carbon (SOC) might affect the mobility of Se in soil. Rather than including it with other such soil variables (near iv and v in the list) it was inserted low in the sequence because of the moderate correlation (0.68) between SOC and TMAH-extractable Se already included at position iii.

   ix. SOC

6. It was suggested speculatively that the soil-bound P might be an indicator of general anion binding capacity of the soil

   x. Oxalate P

   xi. PBI

S.6 Ordering of environmental covariates as potential predictors of Se concentration in grain

The selected order of environmental covariates for spatial prediction of Se concentration in grain is shown in Table 2. The rationale for this ordering is summarized below.

1. Climatic variables. These were selected as likely to be the most useful predictors because of the expected effects of climate on Se content in the soil due both to deposition in precipitation, and the loss of Se from the soil due to leaching (Jones et al., 2017).

i. Precipitation

ii. Temperature

2. Terrain variables. In the Ethiopian highlands landscape position has been recognized as a key source of variation in soil nutrient status and response to management (e.g. Tamene et al, 2017; Tesfahunegn *et al.*, 2011) reflecting erosional and depositional processes. For this reason slope and topographic index were considered next.

   iii. Slope

   iv. Topographic index

3. Vegetation vigour. This was included late in the set of predictors, as less likely to be useful than the physical factors. However, it was considered that vegetation vigour might reflect both the return of Se to the soil in organic forms, released into the soil on mineralization, and possibly the enhanced interception of Se in atmospheric dust and droplets on leaf surfaces (Kabata-Pendias, 2011).

   v. MODIS enhanced vegetation index (EVI).

4. The available MODIS Bands were included last. Band MB7 was considered first because it does not appear in the EVI, and the remaining values were then considered in arbitary order.

   vi MODIS 7

   vii MODIS 1

   viii MODIS 2

   ix MODIS 3

References.

Diggle, P. J., Ribeiro, P. J. 2006. Model-Based Geostatistics. Springer, New York

Jones, G.D., Droz, B., Greve, P., Gottschalk, P., Poffet, D., McGrath, S.P., Seneviratne, S.I., Smith, P., Winkel, L.H.E. 2017. Selenium deficiency risk predicted to increase under future climate change. PNAS, 114, 2848–2853.

Journel, A.G., Huijbregts, Ch.J. 1978. Mining Geostatistics. Academic Press, London.

Kabata-Pendia, A. 2011. Trace elements in soil and plants. 4th Edition. CRC Press, Boca Raton.

Lark, R.M. 2009. Kriging a soil variable with a simple non-stationary variance model. Journal of Agricultural Biological and Environmental Statistics, 14, 301–321.

Marchant, B.P., Lark, R.M. 2007. Estimating linear models of coregionalization by residual maximum likelihood. European Journal of Soil Science, 58, 1506–1513.

Marchant, B.P., Newman, S., Corstanje, R., Reddy, K.R., Ozborne, T.Z., Lark, R.M. 2009. Spatial monitoring of a non-stationary soil property: Phosphorus in a Florida water conservation area. European Journal of Soil Science, 60, 757–769.

Orton, T.G., Pringle, M.J., Bishop, T.F., Paige, K.L., Dalala, R.C. 2014. Spatial prediction of soil organic carbon stock using a linear model of coregionalisation. Geoderma, 230-231, 119–130.

Stein, M.L. 1999. Interpolation of spatial data, some theory for kriging. Springer, New York.

Tamene, L., Amede, T., Kihara, J., Tibebe, D., Schulz, S. 2017. A review of soil fertility management and crop response to fertilizer application in Ethiopia: Towards development of site- and context-specific fertilizer

recommendation. CIAT Publication No. 443. International Center for Tropical Agriculture (CIAT), Addis Ababa, Ethiopia. 86 p. Available at: http://hdl.handle.net/10568/82996

Tesfahunegn, G.B., Tamene, L., Vlek, P.L.G. 2011. Catchment-scale spatial variability of soil properties and implications on site-specific soil management in northern Ethiopia. Soil and Tillage Research, 117, 124–139.

Verbeke, G., Molenberghs, G. 2000. Linear mixed models for longitudinal data. Springer-Verlag, New York.

Webster, R., Oliver, M.A. 2007. Geostatistics for Environmental Scientists. 2nd Edition John Wiley & Sons, Chichester.

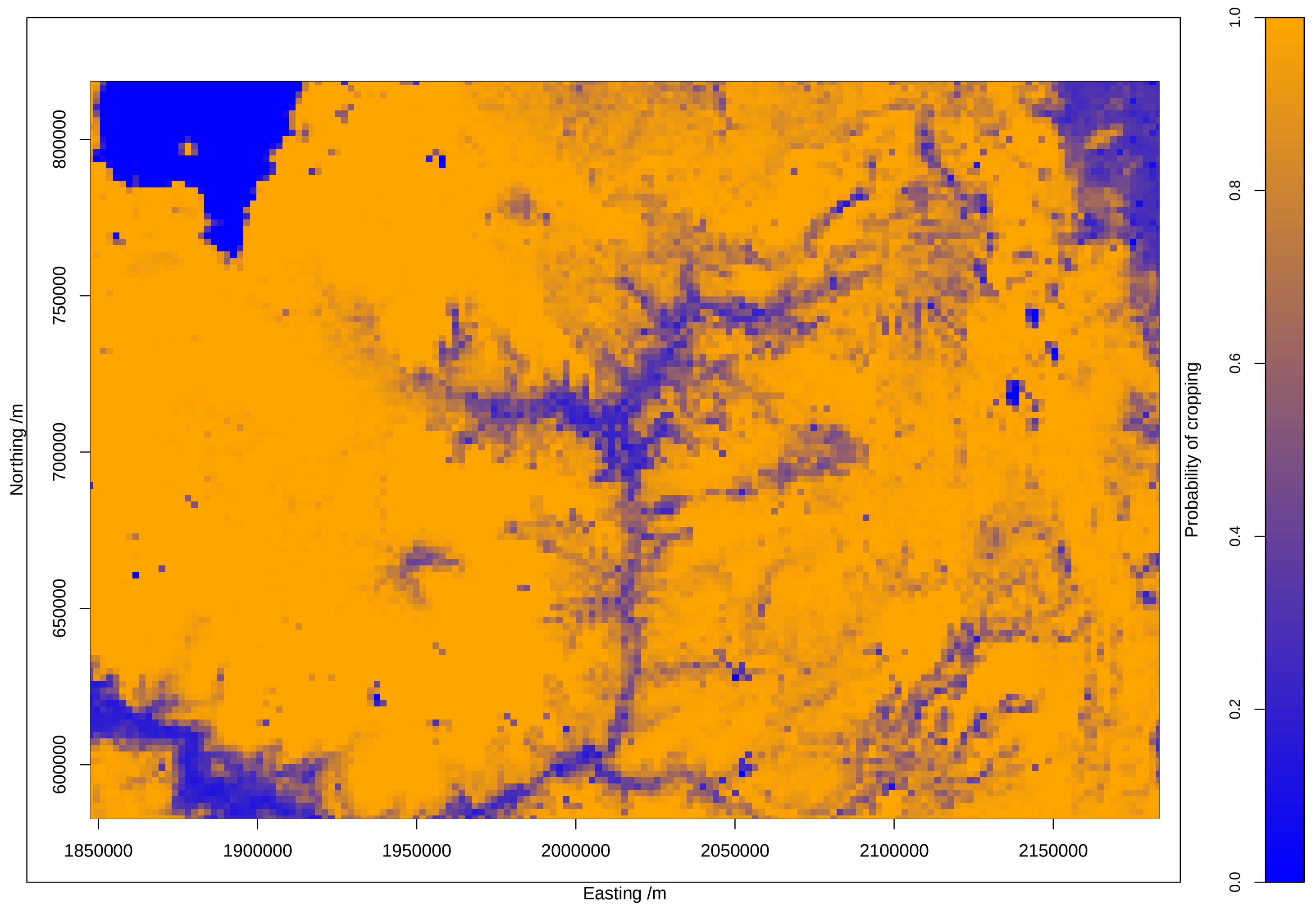

Figure S1: Probability that arable cropping is found at nodes on a 500 m grid over the sample region. Coordinates are in metres relative to datum Latitude 5 degrees N, Longitude=20 degrees E on the Lambert Azimuthal Equal Area projection.

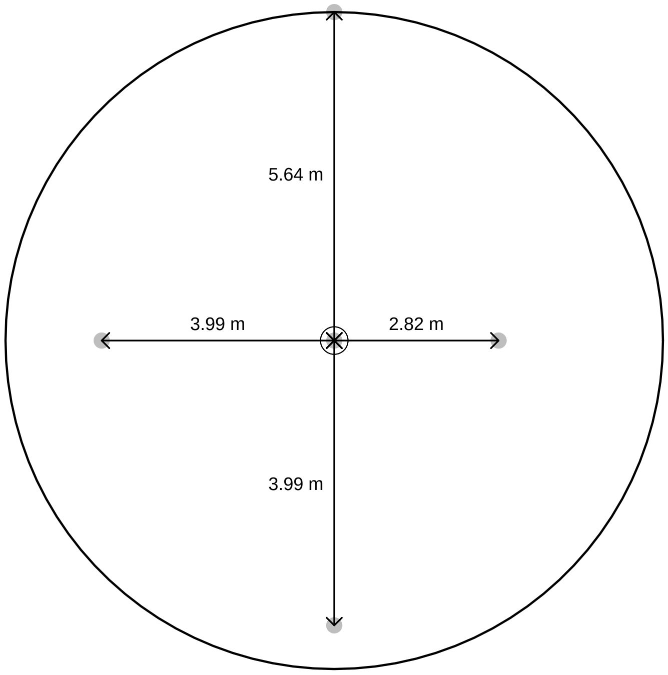

Figure S2: Target layout of five sample points (grey circles)

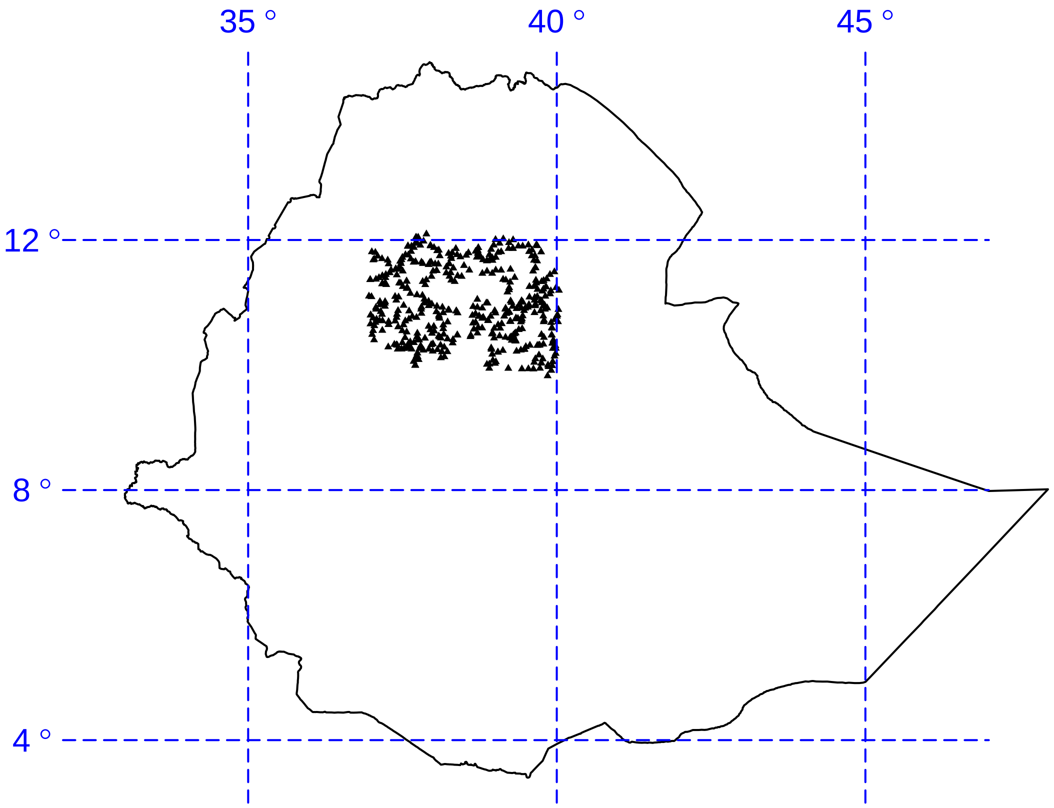

Figure S3: Location of sample sites (black triangles) relative to the borders of Ethiopia (black lines). Degrees longitude (East, WGS84) and latitude (North) are shown as vertical and horizontal dashed lines. The boundary of Ethiopia is taken from file `gadm36_ETH_0_sp.rds`, provided by GADM, `https://gadm.org/index.html`, and reproduced under the terms of the GADM licence `https://gadm.org/license.html` The boundaries, denominations, and any other information shown on this map do not imply any judgment about the legal status of any territory, or constitute any official endorsement or acceptance of any boundaries, on the part of any Government.

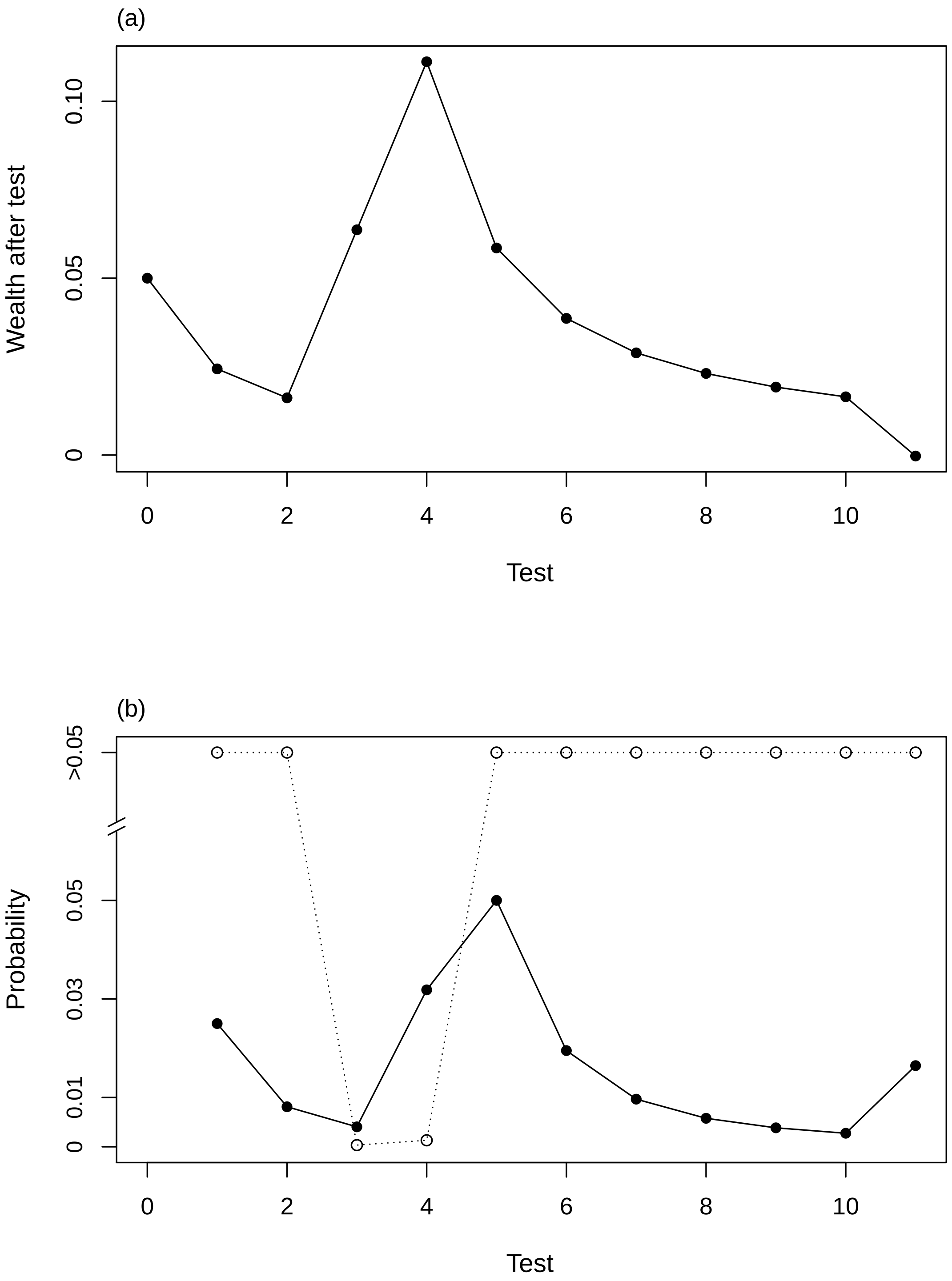

Figure S4: Ordered tests for site (soil) variable selection, wheat grain Se. The sequence of predictors is as given in Table 2. The graph at the top (a) shows the α-wealth over the sequence of tests and the lower graph (b) shows the p-values for successive tests (open symbols) and the corresponding threshold values with marginal false discovery rate control

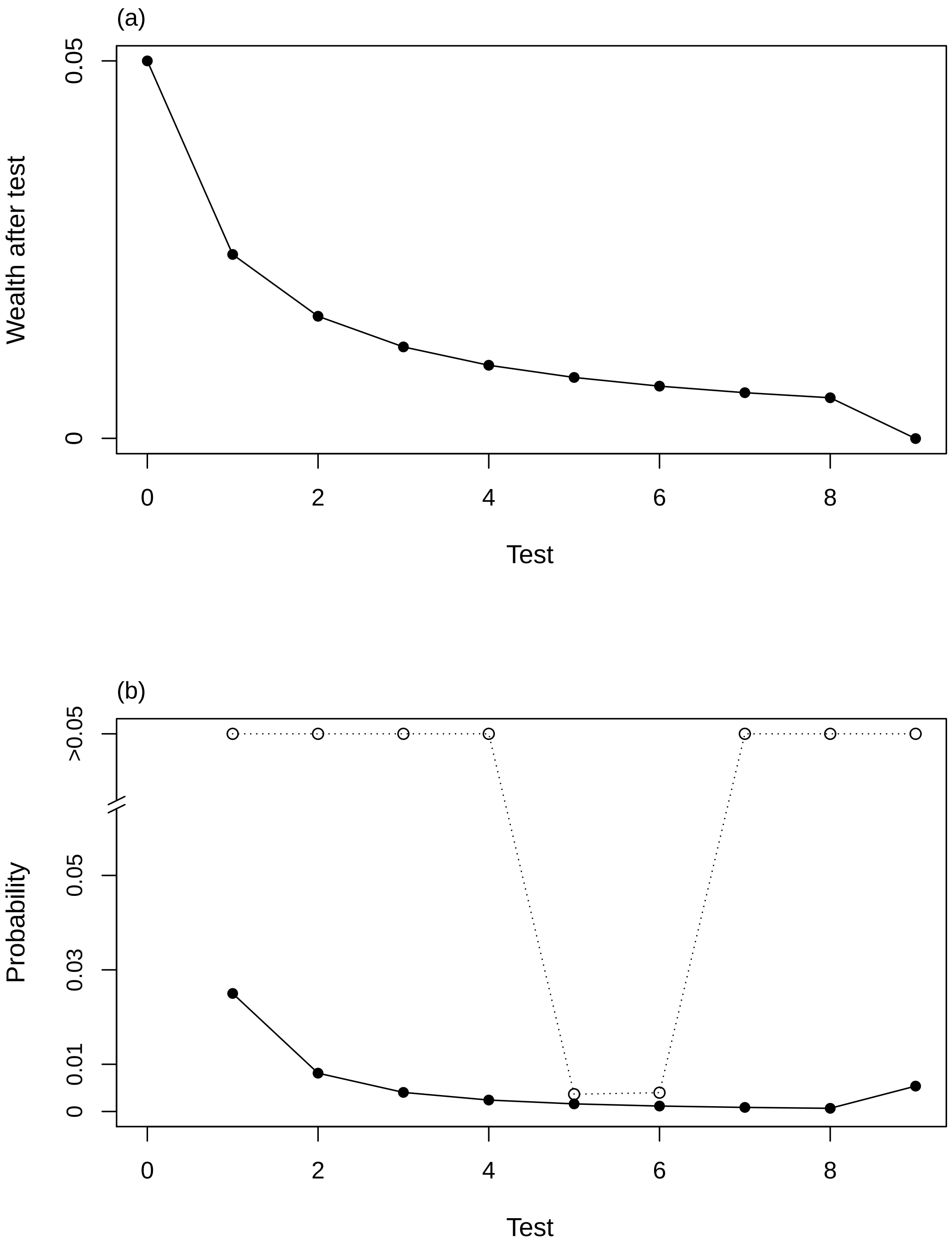

Figure S5: Ordered tests for covariate selection, wheat Se. The sequence of predictors is as given in Table 2. The graph at the top (a) shows the α-wealth over the sequence of tests and the lower graph (b) shows the p-values for successive tests (open symbols) and the corresponding threshold values with marginal false discovery rate control

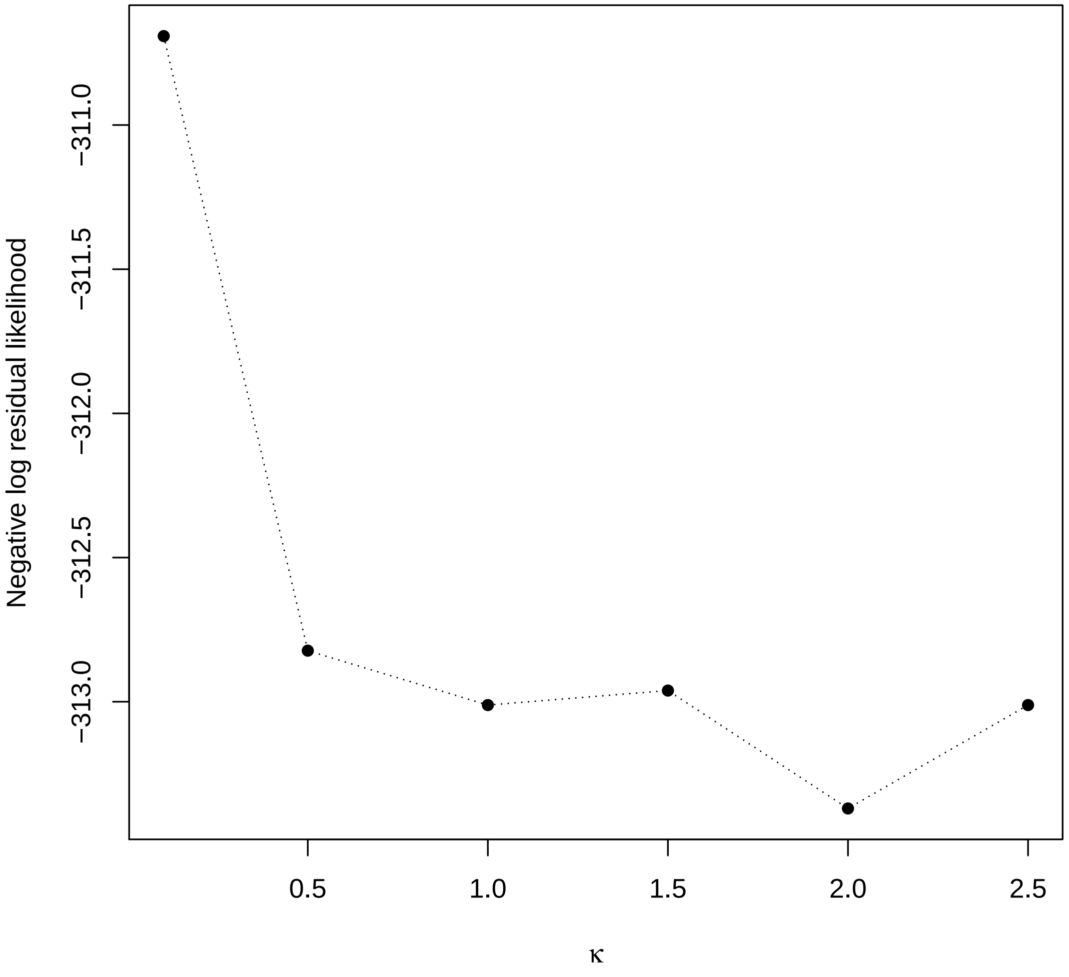

Figure S6: Profile likelihood on κ for teff Se LMCR.

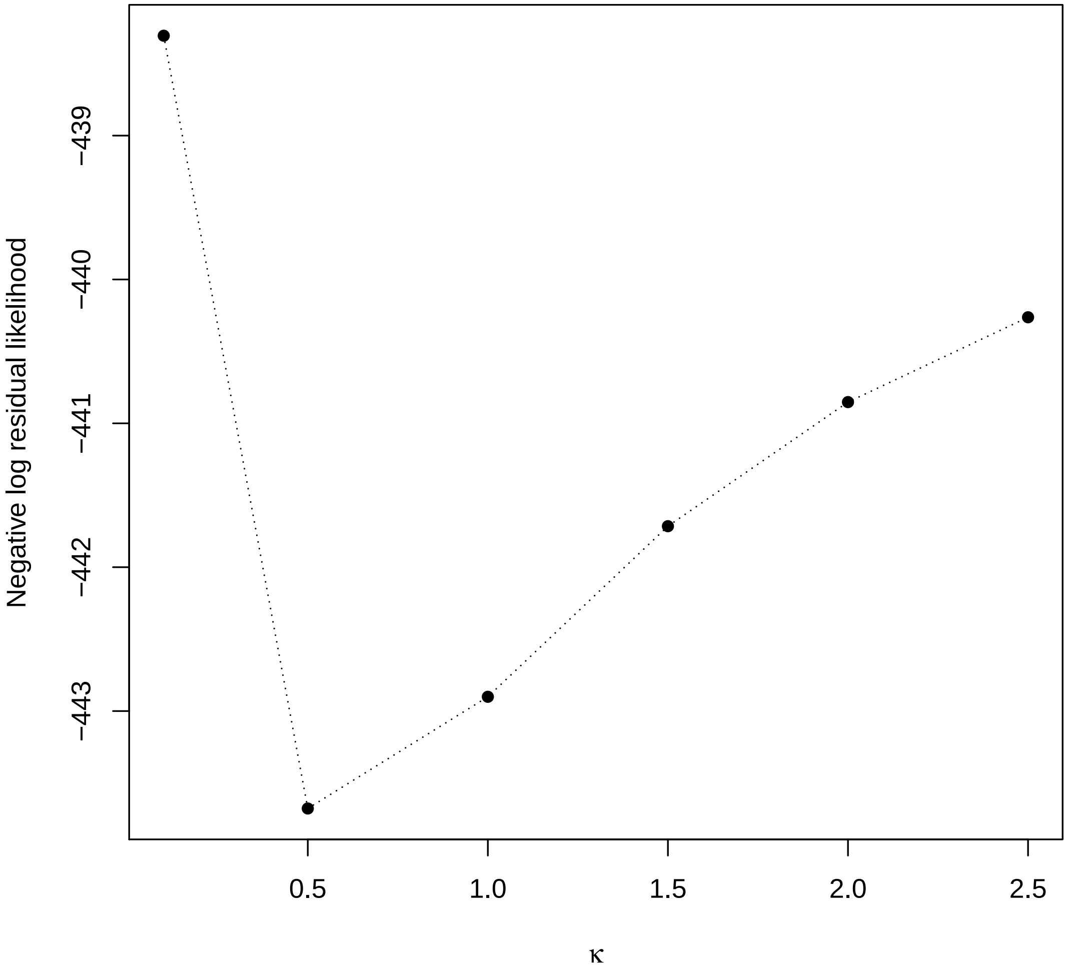

Figure S7: Profile likelihood on the κ parameter (abscissa) for wheat Se LMCR.

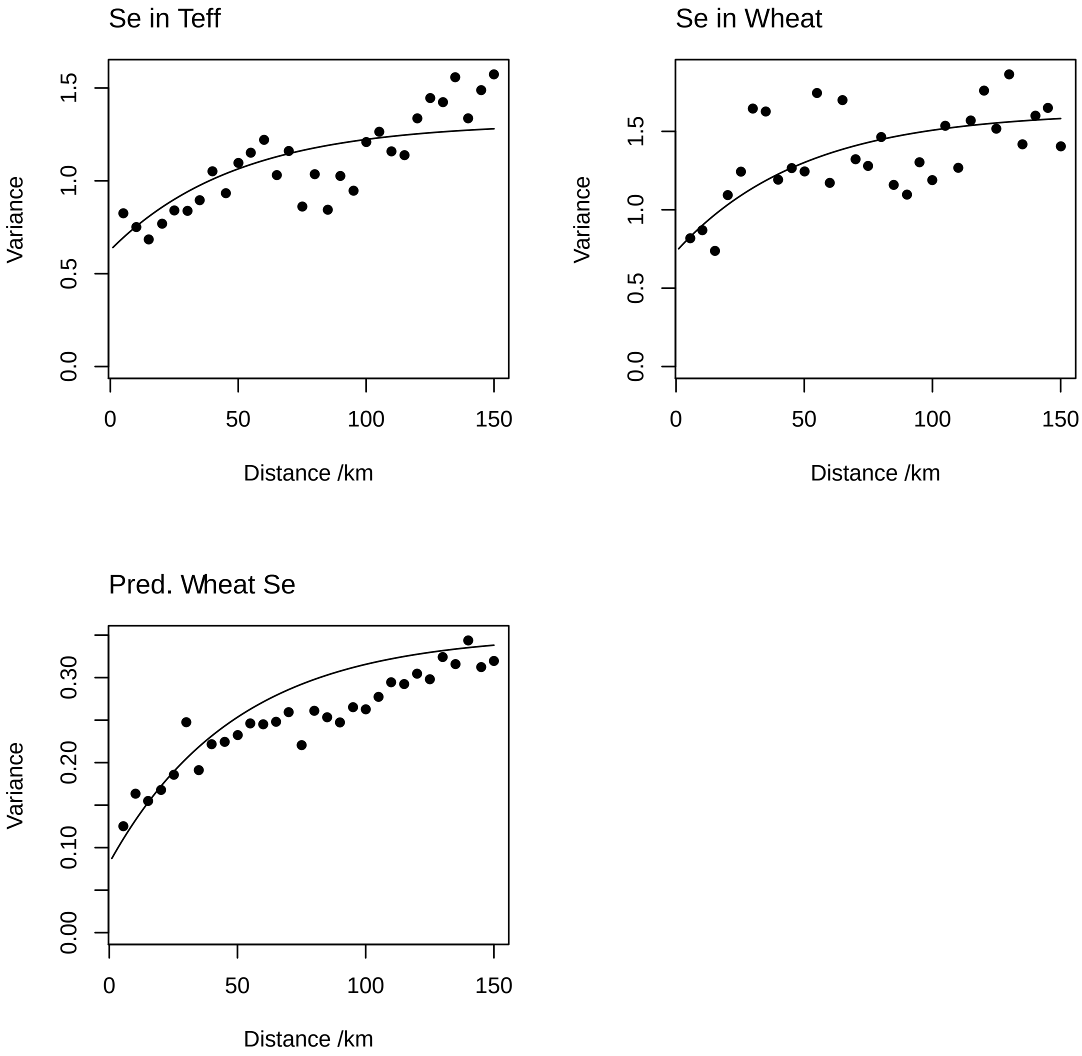

Figure S8: Autovariograms from wheat Se LMCR.

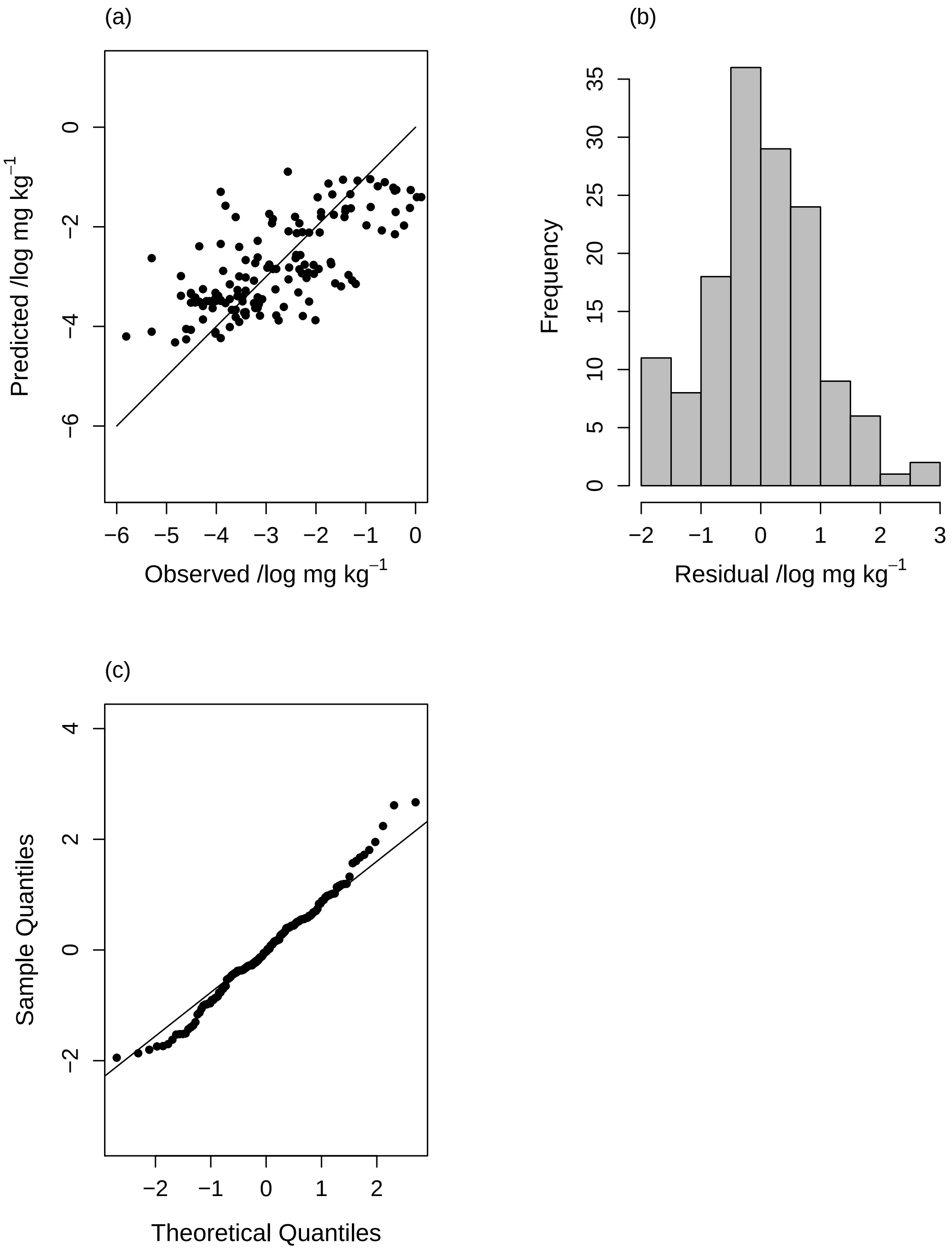

Figure S9: Cross-validation plots (OK) for teff Se LMCR.

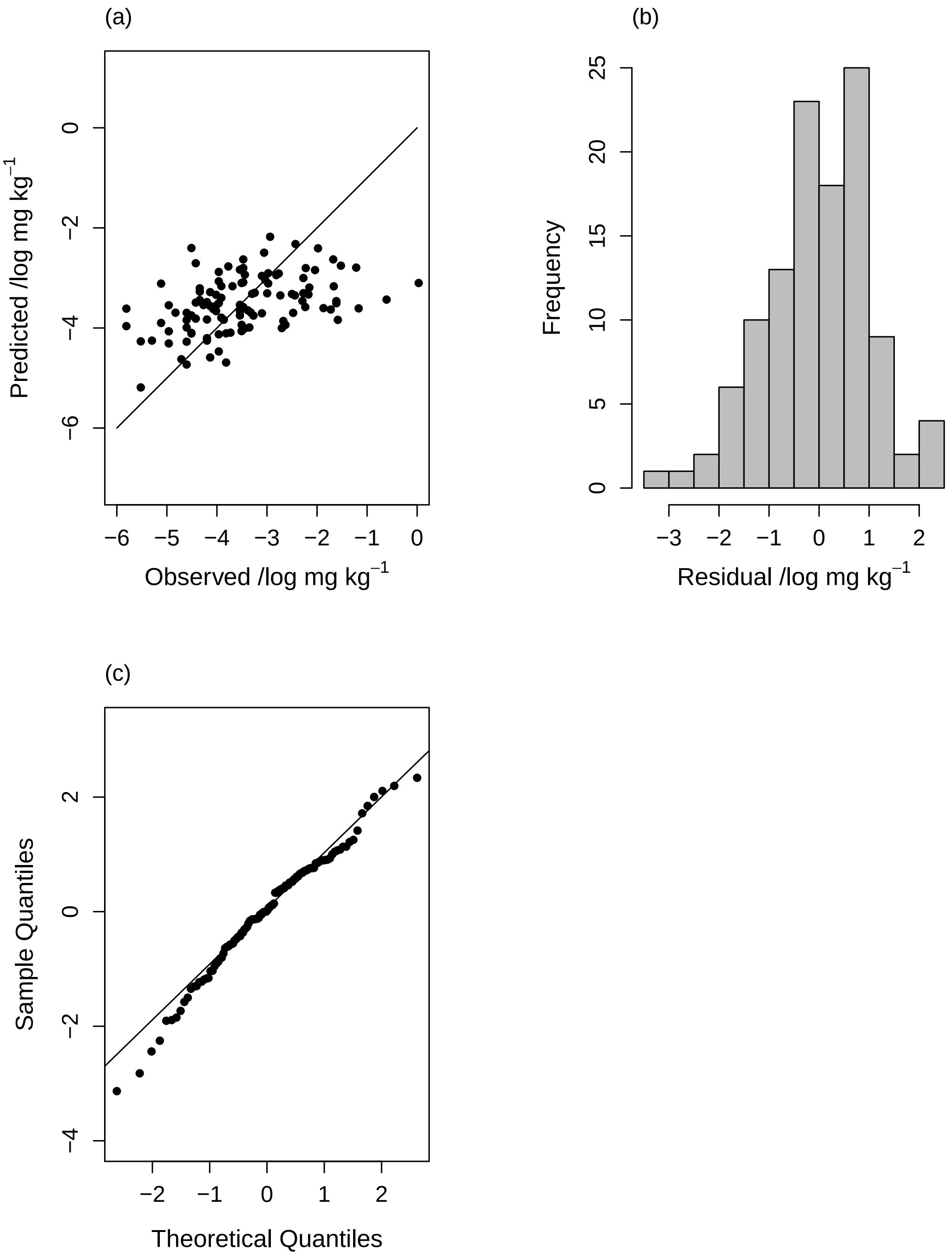

Figure S10: Cross-validation plots (OK) for wheat Se LMCR.
